# Discovery and Validation of Novel Biomarkers for Colorectal Neoplasia Detection via Plasma Metabolomics

**DOI:** 10.1002/mco2.70201

**Published:** 2025-06-06

**Authors:** Jianv Huang, Le Wang, Xiang Zhang, Xinyi Liu, Junyan Miao, Yuefan Shen, Chengqu Fu, Xianxiu Ge, Xue Wang, Jiancong Hu, Guanman Li, Yang Sun, Yinglei Miao, Juncheng Dai, Lingbin Du, Hongxia Ma, Guangfu Jin, Ni Li, Lin Miao, Zhibin Hu, Xiaosheng He, Jun Yu, Hongbing Shen, Dong Hang

**Affiliations:** ^1^ Department of Epidemiology Jiangsu Key Lab of Cancer Biomarkers, Prevention and Treatment, Collaborative Innovation Center for Cancer Personalized Medicine School of Public Health Nanjing Medical University Nanjing China; ^2^ Zhejiang Provincial Office for Cancer Prevention and Control, Zhejiang Cancer Hospital, Institute of Basic Medicine and Cancer (IBMC), Chinese Academy of Sciences Hangzhou China; ^3^ Institute of Digestive Disease Department of Medicine and Therapeutics State Key Laboratory of Digestive Disease Li Ka Shing Institute of Health Sciences CUHK Shenzhen Research Institute The Chinese University of Hong Kong Hong Kong SAR China; ^4^ Medical Centre for Digestive Diseases The Second Affiliated Hospital of Nanjing Medical University Nanjing China; ^5^ Department of Colorectal Surgery Guangdong Provincial Key Laboratory of Colorectal and Pelvic Floor Diseases Guangdong Institute of Gastroenterology The Sixth Affiliated Hospital of Sun Yat‐sen University Guangzhou China; ^6^ Department of Gastroenterology The First Affiliated Hospital of Kunming Medical, University Kunming China; ^7^ Office of Cancer Screening National Cancer Center/National Clinical Research Center for Cancer/Cancer Hospital, Chinese Academy of Medical Sciences and Peking Union Medical College Beijing China

**Keywords:** biomarkers, colorectal adenoma, colorectal cancer, early diagnosis, metabolomics

## Abstract

Metabolic disturbance plays a critical role in the initiation of colorectal cancer (CRC), yet the identification of metabolites that are useful for early detection of CRC and its precursor lesions remains elusive. We conducted an untargeted plasma metabolomic profiling by liquid chromatography‐mass spectrometry in a two‐stage case–control study, including 219 CRC cases, 164 colorectal adenoma (CRA) cases, and 219 normal controls (NC) as a training set, and 91 CRC, 115 CRA, and 109 NC as a validation set. Among 891 named metabolites, 239 were significantly altered in CRC versus NC, 26 in CRA versus NC, and 88 in CRC versus CRA within the training set. The results were stable when adjusting for potential confounders. A panel of 10 metabolites, including six lipid species, one benzenoid, one organoheterocyclic compound, one organic acid derivative, and one organic oxygen compound, showed optimal performance in discriminating CRC from NC (AUC = 0.81) in the validation. Moreover, a panel of seven metabolites exhibited optimal performance in discriminating CRA from NC, with an AUC of 0.89. Our findings provide novel evidence supporting specific plasma metabolites, particularly those implicated in lipid metabolism, as promising biomarkers for the early detection of CRC.

## Graphical abstract

We identified novel and reproducible metabolic biomarkers for colorectal cancer and adenoma. Plasma metabolites, particularly those involved in lipid metabolism, may serve as non‐invasive biomarkers for early detection of colorectal cancer.

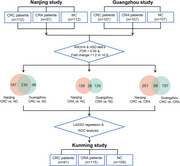

## 1 Introduction

Globally, colorectal cancer (CRC) ranks third in incidence among all malignancies and is the second most common cause of cancer mortality, with an estimated 1.9 million newly diagnosed cases and 0.9 million deaths in 2022 [1]. While highly developed countries have seen a decrease in the burden of CRC, primarily due to national CRC screening programs, low‐ and middle‐income countries have experienced a rapid upward trend [1]. In China, CRC is a rapidly increasing and deadly disease, accounting for 28.8% of all incident CRC cases and 30.6% of all CRC‐caused deaths worldwide [2]. Unfortunately, less than 20% of the cases were diagnosed at stage I, with approximately half being diagnosed at stages III and IV [3].

Although colonoscopy is widely acknowledged as the gold standard for detecting CRC, its limited adherence and screening effectiveness in the general population present significant challenges [4, 5]. Pre‐selecting individuals who benefit the most from screening has been proposed as great potential for enhancing the cost‐effectiveness of screening programs [6, 7]. Fecal hemoglobin measured through the fecal immunochemical test (FIT) is the widely used biomarker to facilitate such pre‐selection. According to a previous meta‐analysis, the sensitivity of FIT in asymptomatic average‐risk adults was 91% for CRC and 40% for advanced adenoma at a positivity threshold of 10 µg hemoglobin/g [8]. Despite its safety, affordability, and ease of use, the poor sensitivity of FIT for detecting adenomas has limited its potential to further decrease the incidence of CRC [9, 10].

Recent studies have emphasized the potential of single biomarker and biomarker combinations in advancing blood‐based tests for CRC screening [11]. Carcinoembryonic antigen (CEA) serves as a widely employed clinical marker for CRC diagnosis [11]. However, the diagnostic sensitivity of CEA was only 33% for early‐stage CRC and 14% for colorectal adenoma [12, 13]. Other biomarkers, such as serum cancer antigen 19‐9, cytokeratin fragment 21‐1 (CyFra21‐1), high‐sensitivity C‐reactive protein (hs‐CRP), and ferritin, have also been investigated, with individual areas under the curve (AUC) all less than 0.65 [13, 14]. A combination of CEA, CyFra21‐1, ferritin, and hs‐CRP could improve the performance for detecting CRC and high‐risk adenoma, but the sensitivity (60%) and specificity (75%) remained suboptimal [14]. These findings underscore the urgent necessity for more sensitive and specific biomarkers to enhance early detection of CRC and its precursors.

Metabolic reprogramming is a recognized core hallmark of cancer development [15]. Metabolomics, an advanced technology that can measure a broad spectrum of metabolites, offers a new opportunity to discover novel biomarkers for early cancer detection and to decipher mechanisms underlying carcinogenesis [16]. Several well‐designed studies have reported promising diagnostic accuracy for CRC using a combination of serum tyrosine and glutamine‐leucine [17], as well as different panels of serum metabolite derived from the gut microbiome [18, 19], with the AUCs of the receiver operating characteristic curve (ROC) ranging from 0.80 to 0.98. However, the metabolites identified in different studies had poor overlap and the diagnostic performance for adenoma varied significantly [20]. These discrepancies are likely due to the heterogeneity of study populations, such as age, sex, lifestyle factors, and disease status, as well as variations in metabolomic methods including analytical instruments, metabolome coverage, and data processing. Therefore, further high‐quality research is crucial to identify and validate reliable biomarkers that can facilitate CRC early detection.

In this study, we initially carried out two matched case–control studies serving as a training set, in which untargeted metabolomics profiling of plasma samples was performed and pairwise comparisons between CRC, colorectal adenoma (CRA), and normal control (NC) were conducted to identify reproducible metabolic biomarkers. Subsequently, we utilized machine learning approaches to develop metabolic models and externally validated their diagnostic performance for CRC and CRA in another independent case–control study.

## 2 Results

### 2.1 Metabolic Profiles in CRC Progression

**FIGURE 1:**
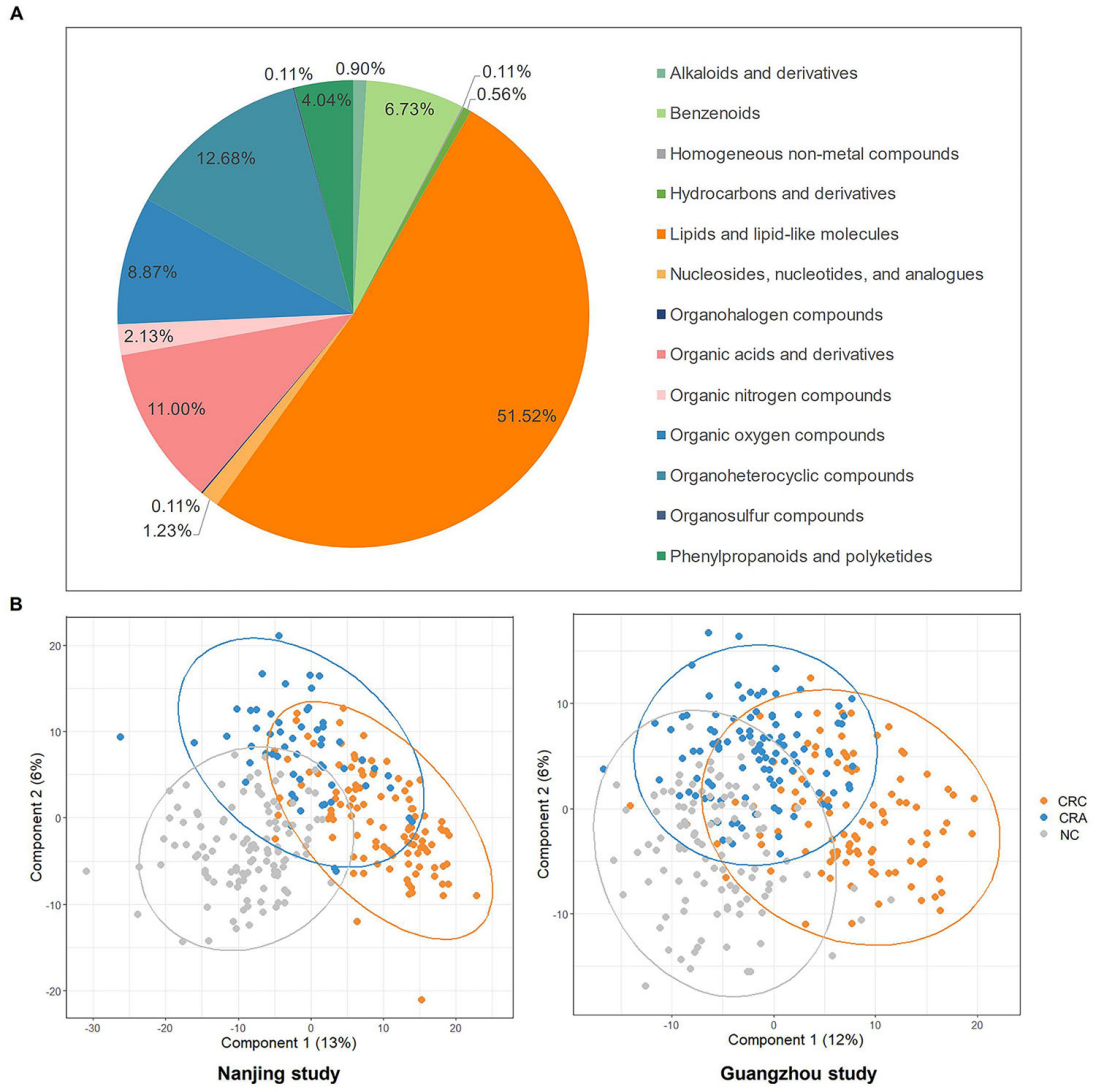
Untargeted metabolic profiles in the Nanjing and Guangzhou studies. (A) A pie graph of 13 chemical superclasses of 891 plasma metabolites. (B) PLS‐DA plots based on 891 metabolites revealed clear separation of CRC, CRA, and NC groups in the Nanjing and Guangzhou studies. CRA, colorectal adenoma; CRC, colorectal cancer; NC, normal control; PLS‐DA, partial least squares discriminant analysis.

A total of 891 plasma metabolites were included in the analysis and grouped into 13 chemical superclasses. These superclasses included lipids and lipid‐like molecules (51.52%), organoheterocyclic compounds (12.68%), organic acids and derivatives (11%), organic oxygen compounds (8.87%), benzenoids (6.73%), phenylpropanoids and polyketides (4.04%), organic nitrogen compounds (2.13%), nucleosides, nucleotides, and analogues (1.23%), alkaloids and derivatives (0.90%), hydrocarbons and derivatives (0.56%), homogeneous non‐metal compounds (0.11%), organohalogen compounds (0.11%), and organosulfur compounds (0.11%) (Figure 1A). Partial least squares‐discrimination analysis (PLS‐DA) revealed significant differences in the overall metabolomic profiles for CRC, CRA, and NC in both the Nanjing and Guangzhou studies (*p* < 0.05) (Figure 1B).

### 2.2 Identification of Metabolic Biomarkers for Colorectal Neoplasia

**FIGURE 2:**
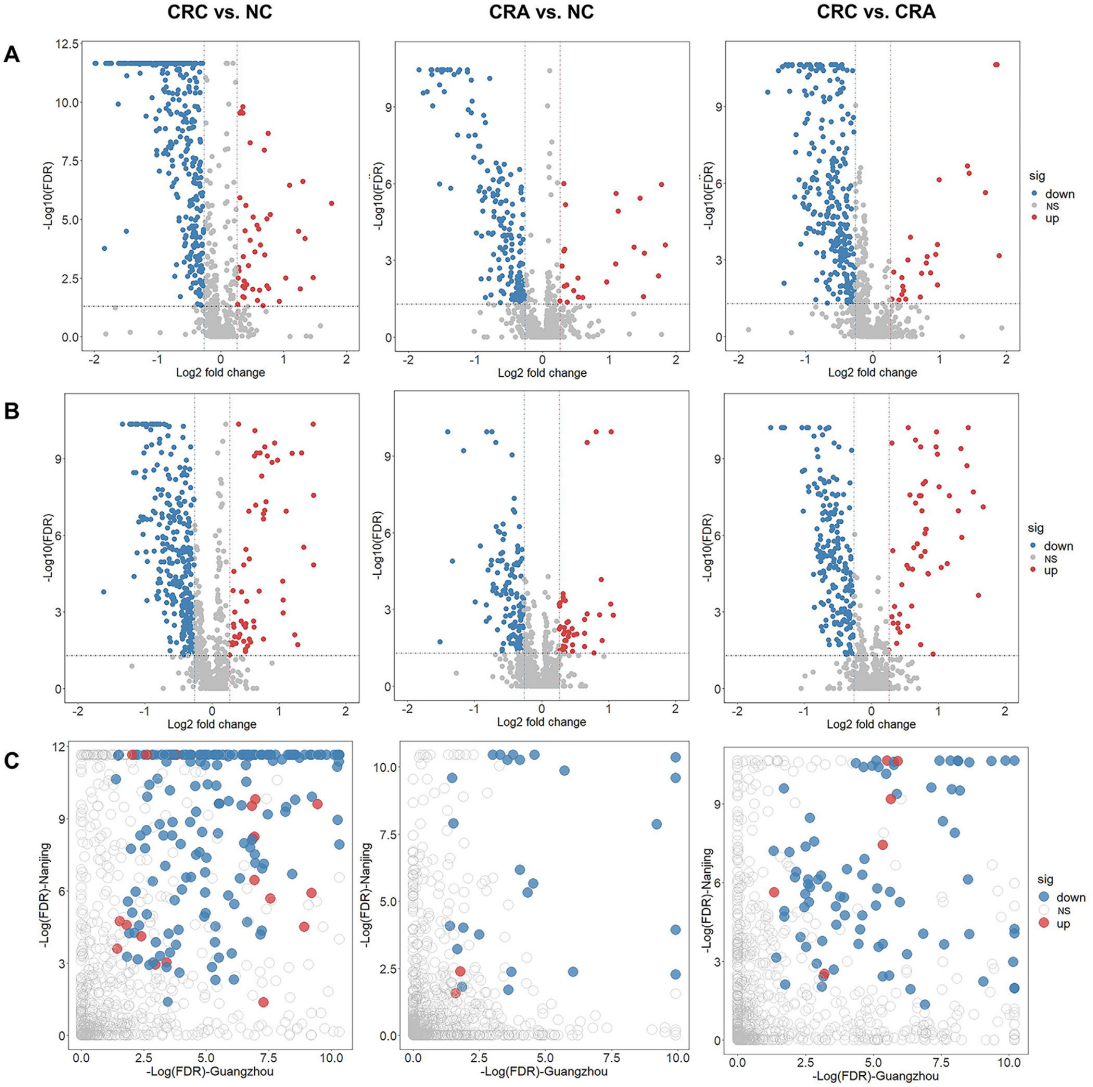
Pairwise comparisons of plasma metabolites between CRC, CRA, and NC in the Nanjing and Guangzhou studies. Volcano plots of one‐way ANOVA and Tukey's HSD test results in the Nanjing (A) and Guangzhou (B) studies. The vertical dashed lines indicate the fold changes of 0.8 (left) and 1.2 (right). The horizontal dashed line indicates the FDR of 0.05. (C) Scatter plots for the consistency of differential metabolites between the two studies. Each point represents a metabolite, with significant changes depicted in red (upregulated) or blue (downregulated), and all other metabolites shown in grey. ANOVA, one‐way analysis of variance; CRC, colorectal cancer; CRA, colorectal adenoma; FDR, false discovery rate; HSD, honestly significant difference; NC, normal control.

We identified various metabolites that had significantly altered abundances between CRC, CRA, and NC (fold change [FC] > 1.2 or < 0.8, and *p*_FDR_ < 0.05). Volcano plots were generated to display the log_2_ fold change (*x*‐axis) versus the adjusted −log_10_ *p* value from the Tukey's honestly significant difference (HSD) test (*y*‐axis) for each metabolite in Nanjing (Figure 2A) and Guangzhou (Figure 2B). After cross‐validating the results of the two studies (Figure 2C, Table S1), we detected 239 metabolites with significant differences in CRC patients compared to NC, including 19 elevated metabolites (six lipids and lipid‐like molecules, five organic acids and derivatives, four organic oxygen compounds, two organoheterocyclic compounds, one benzenoid, and one organic nitrogen compound) and 220 reduced metabolites (164 lipids and lipid‐like molecules, 15 benzenoids, 9 organoheterocyclic compounds, 7 organic acids and derivatives, 5 phenylpropanoids and polyketides, 14 organic oxygen compounds, 4 nucleosides, nucleotides, and analogues, 1 alkaloids and derivative, and 1 organic nitrogen compound). Differential metabolites between CRC and NC were illustrated by the heatmaps (Figures S1–S3).

By comparing CRA with NC, we identified 26 differential metabolites, including two elevated metabolites (one hydrocarbon and one organoheterocyclic compound) and 24 reduced metabolites (15 lipids and lipid‐like molecules, 4 organoheterocyclic compounds, 2 organic acids and derivatives, 1 benzenoid, 1 organic oxygen compound, and 1 organic nitrogen compound) (Table S1). Notably, the levels of 15 metabolites were consistently lower in both CRA and CRC as compared to NC (Figure S4). The heatmaps were also generated to show differential metabolites between CRA and NC (Figures S1–S3).

By comparing CRC with CRA, a total of 88 metabolites showed significant differences, including six elevated metabolites (three organic oxygen compounds, two lipids and lipid‐like molecules, and one organoheterocyclic compound) and 82 reduced metabolites (52 lipids and lipid‐like molecules, 12 organic oxygen compounds, 6 benzenoids, 4 organoheterocyclic compounds, 4 organic acids and derivatives, 2 nucleosides, nucleotides, and analogues, and 2 phenylpropanoids and polyketides). The differential metabolites were also depicted in the heatmaps (Figures S1–S3). The multivariate logistic regression models with adjustment for age, area, body mass index, smoking status, and alcohol drinking confirmed the robustness of the results (Table S1).

**FIGURE 3:**
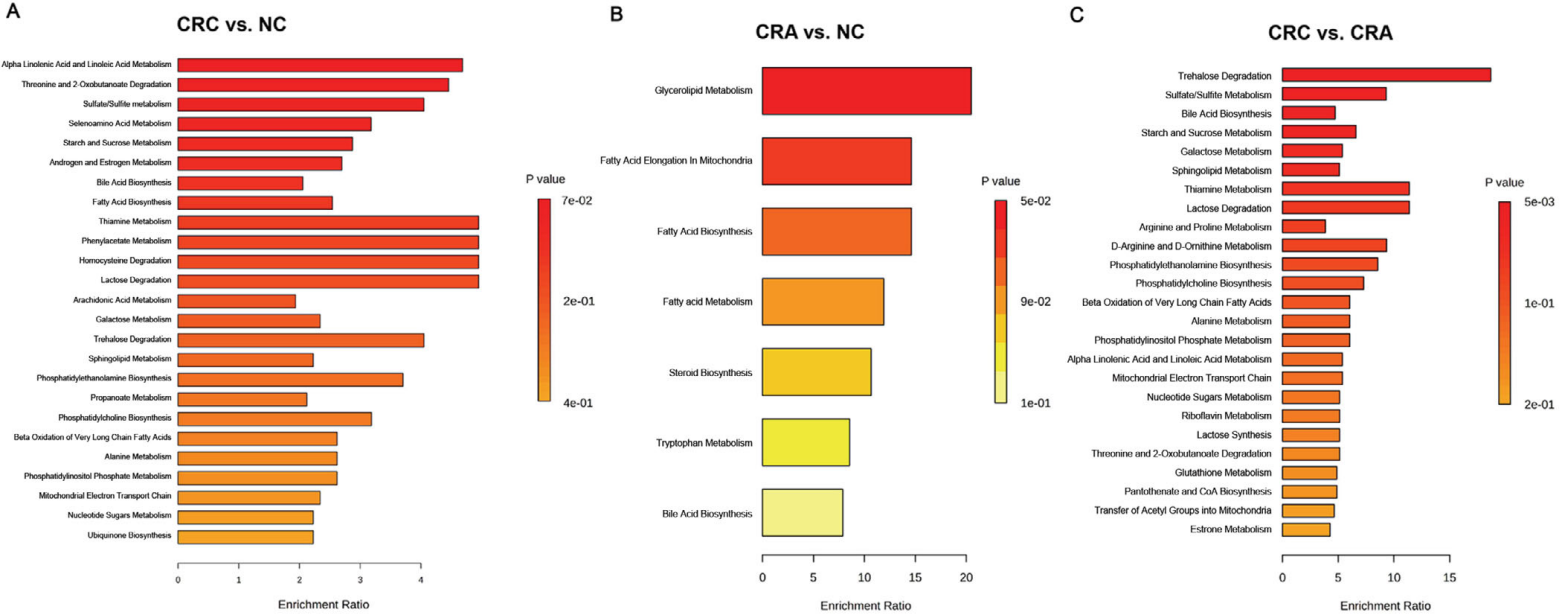
Pathway enrichment analysis for CRC versus NC (A), CRA versus NC (B), and CRC versus CRA (C). CRA, colorectal adenoma; CRC, colorectal cancer; NC, normal control.

We performed a pathway enrichment analysis to deepen our insight into the functional roles of metabolites that showed significant differences. The top three significant pathways for CRC versus NC were alpha linolenic acid and linoleic acid metabolism, threonine and 2‐oxobutanoate degradation, and sulfate/sulfite metabolism (Figure 3A). The differential metabolites within the top three pathways are shown in Table S2. The pathway of alpha linolenic acid and linoleic acid metabolism (normalized enrichment score [NES], −1.36; *p* = 0.034) was found to be downregulated in the CRC compared to the NC (Figure S5). Within the pathway, the levels of stearidonic acid and linoleic acid were significantly lower in CRC than in NC. In the case of CRA versus NC, the top three significant pathways were glycerolipid metabolism, fatty acid (FA) elongation in mitochondria, and FA biosynthesis (Figure 3B). The pathway of gycerolipid metabolism (NES, −1.39; *p* = 0.046) was found to be downregulated in CRA compared to NC. The level of palmitic acid was significantly lower in CRA than in NC (Figure S5). For CRC versus CRA, the top three significant pathways were trehalose degradation, sulfate/sulfite metabolism, and bile acid biosynthesis (Figure 3C). In the trehalose degradation pathway (NES, 1.22; *p* = 0.286), the level of trehalose was significantly higher, while the level of adenosine diphosphate was significantly lower in CRC than in CRA (Figure S5).

### 2.3 Assessment of Diagnostic Performance for Colorectal Neoplasia

**FIGURE 4:**
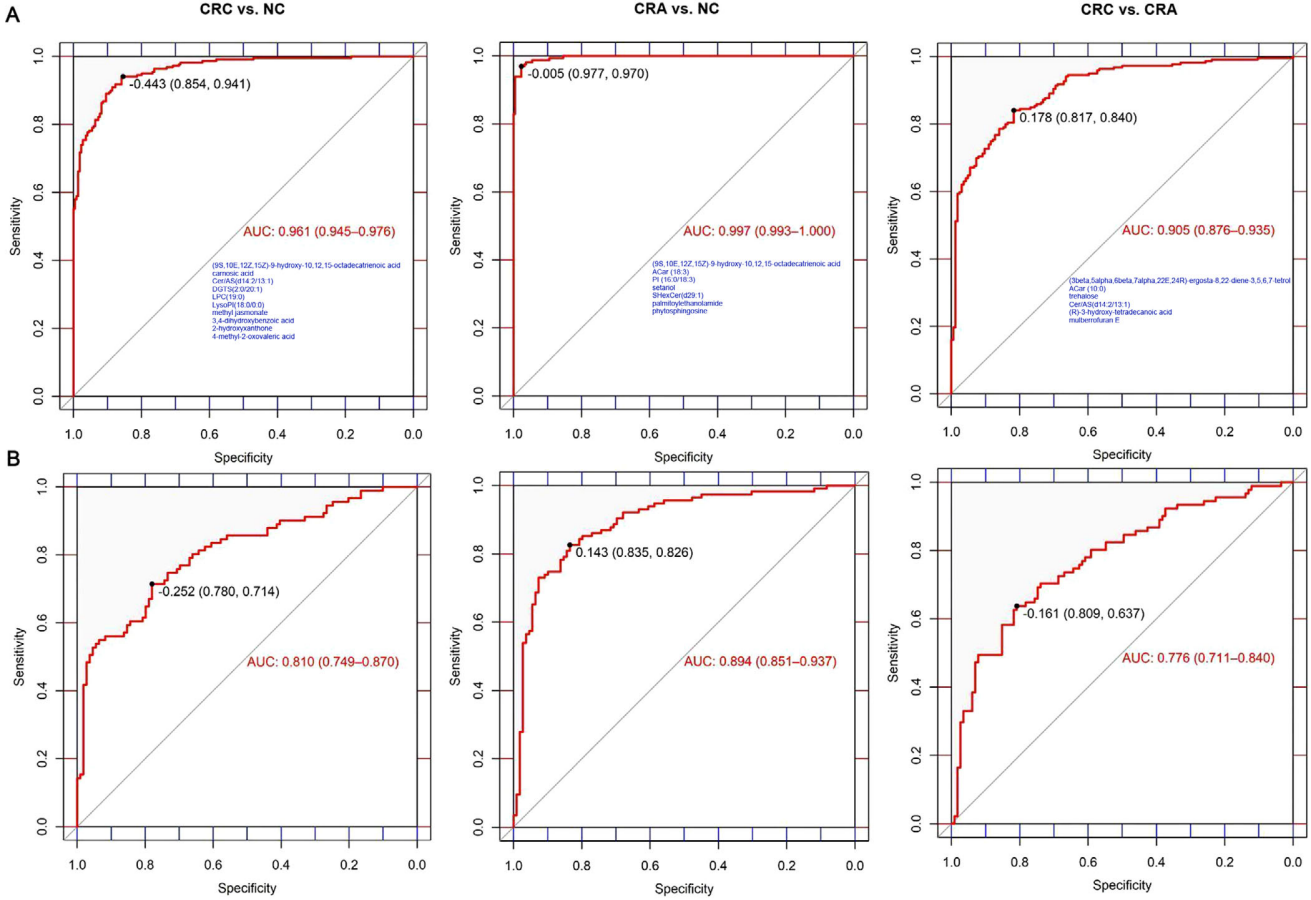
Receiver operator characteristic analysis of metabolic biomarkers in the training (A) and validation (B) sets. The resulting models consisted of 10 metabolites for discriminating CRC from NC, seven metabolites for discriminating CRA from NC, and six metabolites for discriminating CRC from CRA. AUC, area under the receiver operating characteristic curve; CRC, colorectal cancer; CRA, colorectal adenoma; NC, normal control.

**FIGURE 5:**
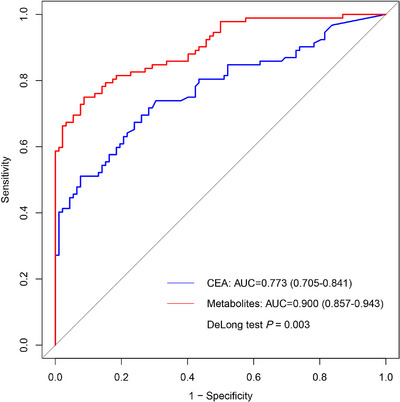
Receiver operating characteristic curves for plasma metabolites and CEA to discriminate CRC from NC. AUC, area under the receiver operating characteristic curve; CEA, carcinoembryonic antigen.

We utilized the least absolute shrinkage and selection operator (LASSO) algorithm to select biomarkers of diagnostic ability among the above‐mentioned differential metabolites. Among the 239 metabolites showing significant differences between CRC and NC, we found that a panel of 10 metabolites, which were downregulated in CRC, displayed an AUC of 0.961 (95% CI: 0.945–0.976; sensitivity: 94.1%, specificity: 85.4%) to discriminate CRC from NC in the training set and an AUC of 0.810 (95% CI: 0.749–0.870; sensitivity: 71.4%, specificity: 78.0%) in the validation set (Figure 4). The positive predictive value (PPV) and negative predictive value (NPV) were 0.866 (95% CI: 0.839–0.940) and 0.935 (95% CI: 0.866–0.962), respectively, in the training set, as well as 0.730 (95% CI: 0.646–0.965) and 0.766 (95% CI: 0.664–0.873), respectively, in the validation set (Table 1). In addition, we found that the metabolic panel exhibited significantly superior performance (AUC = 0.900, 95% CI: 0.857–0.943; sensitivity: 75.0%, specificity: 91.3%) in discriminating CRC from NC compared to CEA (AUC = 0.773, 95% CI: 0.705–0.841; sensitivity: 67.4%, specificity: 76.1%) (DeLong test, *p* = 0.003) (Figure 5). The panel included six lipids and lipid‐like molecules ((9S,10E,12Z,15Z)‐9‐hydroxy‐10,12,15‐octadecatrienoic acid, carnosic acid, DGTS(2:0/20:1), LPC(19:0), LysoPI(18:0/0:0), and methyl jasmonate), one benzenoid (3,4‐dihydroxybenzoic acid), one organoheterocyclic compound (2‐hydroxyxanthone), one organic oxygen compound (Cer/AS(d14:2/13:1)), and one organic acid derivative (4‐methyl‐2‐oxovaleric acid). The performance of individual metabolites in discriminating CRC from NC is presented in Table S3, with the AUC ranging from 0.562 to 0.727 calculated by support vector machine (SVM) in the validation set. Moreover, these metabolites together had a good discriminatory ability for early‐stage CRC (0‐II) versus NC (AUC = 0.797, 95% CI: 0.704–0.890) (Figure S6), and displayed similar diagnostic performance for colon cancer (AUC = 0.824, 95% CI: 0.741–0.908) and rectum cancer (AUC = 0.862, 95% CI: 0.794–0.931) in the validation set (Figure S7). Additionally, the panel exhibited an AUC of 0.711 (95% CI: 0.642–0.780) in discriminating CRA from NC and an AUC of 0.854 (95% CI: 0.802–0.907) in discriminating CRC from CRA (Figure S8).

**TABLE 1:** The PPV and NPV of metabolic biomarkers in the training and validation sets.

Datasets	Groups	PPV	NPV
Training set	CRC vs. NC	0.866 (0.839–0.940)	0.935 (0.866–0.962)
	CRA vs. NC	0.970 (0.931–1.000)	0.977 (0.957–1.000)
	CRC vs. CRA	0.860 (0.806–0.959)	0.793 (0.678–0.907)
Validation set	CRC vs. NC	0.730 (0.646–0.965)	0.766 (0.664–0.873)
	CRA vs. NC	0.841 (0.776–0.953)	0.820 (0.727–0.906)
	CRC vs. CRA	0.725 (0.597–0.900)	0.738 (0.658–0.856)

Abbreviations: CRA, colorectal adenoma; CRC, colorectal cancer; NC, normal control; NPV, negative predictive value; PPV, positive predictive value.

We further evaluated the predictive performance of this metabolic panel for four other types of tumors. The panel displayed an AUC of 0.669 (95% CI: 0.564–0.774; sensitivity: 65.5%, specificity: 67.3%) for esophageal cancer, an AUC of 0.663 (95% CI: 0.559–0.767; sensitivity: 41.8%, specificity: 98.2%) for gastric cancer, an AUC of 0.802 (95% CI: 0.716–0.888; sensitivity: 65.5%, specificity: 89.1%) for liver cancer, and an AUC of 0.733 (95% CI: 0.641–0.825; sensitivity: 41.8%, specificity: 94.5%) for breast cancer (Figure S9). The results of low AUCs for the other types of cancer indicate that the panel serves as a relatively specific diagnostic tool for CRC.

When comparing the change in plasma metabolites before and after surgery for CRC patients, we found that among the ten metabolites included in the metabolic panel, the level of 2‐hydroxyxanthone, which was lower in CRC compared to NC, was significantly increased after surgery (Figure S10). Additionally, we compared the levels of selected metabolites in tumor and adjacent normal tissues and found that the level of carnosic acid was significantly decreased in tumor compared to adjacent normal tissues (Figure S11).

Among the 26 differential metabolites between CRA and NC, a panel of seven metabolites was identified to have superior diagnostic performance for CRA. In the training set, these metabolites showed an AUC of 0.997 (95% CI: 0.993–1.000; sensitivity: 97.0%, specificity: 97.7%), a PPV of 0.970 (95% CI: 0.931–1.000), and an NPV of 0.977 (95% CI: 0.957–1.000). The validation set also demonstrated excellent performance, with an AUC of 0.894 (95% CI: 0.851–0.937; sensitivity: 82.6%, specificity: 83.5%), a PPV of 0.841 (95% CI: 0.776–0.953), and an NPV of 0.820 (95% CI: 0.727–0.906) (Figure 4 and Table 1). The seven metabolites were downregulated in CRA, which included five lipids and lipid‐like molecules ((9S,10E,12Z,15Z)‐9‐hydroxy‐10,12,15‐octadecatrienoic acid, ACar (18:3), PI (16:0/18:3), setariol, and SHexCer(d29:1)), one organic acid (palmitoylethanolamide), and one organic nitrogen compound (phytosphingosine).

A panel of six metabolites was identified from the 88 differential metabolites between CRC and CRA to discriminate CRC from CRA. The panel showed an AUC of 0.905 (95% CI: 0.876–0.935; sensitivity: 84.0%, specificity: 81.7%) in the training set and 0.776 (95% CI: 0.711–0.840; sensitivity: 63.7%, specificity: 80.9%) in the validation set (Figure 4). The PPV and NPV were 0.860 (95% CI: 0.806–0.959) and 0.793 (95% CI: 0.678–0.907), respectively, in the training set, as well as 0.725 (95% CI: 0.597–0.900) and 0.738 (95% CI: 0.658–0.856), respectively, in the validation set (Table 1). The six metabolites included three lipids and lipid‐like molecules ((3beta,5alpha,6beta,7alpha,22E,24R)‐ergosta‐8,22‐diene‐3,5,6,7‐tetrol, ACar (10:0), and (R)‐3‐hydroxy‐tetradecanoic acid), two organic oxygen compounds (trehalose and Cer/AS(d14:2/13:1)), and one phenylpropanoid (mulberrofuran E). The ROC analysis based on SVM demonstrated the similar AUC results (Figure S12).

The majority of selected predictors from the random forest remained the same as those from LASSO (Figure S13). Compared to the metabolites selected by LASSO, the metabolites selected by the random forest exhibited similar performance in distinguishing CRC from NC, but inferior performance in distinguishing CRA from NC and CRC from CRA (Figure S14).

## 3 Discussion

In this multicenter case–control study, we identified distinct plasma metabolomic profiles between CRC, CRA, and NC. We also evaluated the diagnostic accuracy of metabolic biomarkers selected by machine learning models for CRC and CRA. The results showed that a panel of 10 metabolites had excellent discrimination ability to distinguish CRC from NC, while seven metabolites performed well in discriminating CRA from NC. Additionally, six metabolites exhibited potential to discriminate CRC from CRA. Most of the metabolites belong to lipids and lipid‐like molecules. Our study thus provides novel evidence indicating that specific lipid metabolites hold significant potential as noninvasive biomarkers for the CRC early detection.

According to a recent systematic review [20], previous studies evaluating the diagnostic potential of metabolomics for CRC were often limited by small sample sizes (typically less than 100 cases) and without replicating their findings in independent datasets. Only a few studies adopted a two‐stage design with discovery and replication phases to increase confidence in the validity of metabolic biomarkers, and even fewer studies have committed to exploring biomarkers for CRA. For instance, based on one hospital, Chen et al. recruited 49 CRC, 12 CRA, and 31 NC cases as a discovery set, as well as 84 CRC, 19 CRA, and 53 NC cases as a validation set. They identified eight gut microbiome‐related serum metabolites with potential for CRC (AUC = 0.92) and CRA (AUC = 0.84) diagnosis in the validation [18]. In addition, Coker et al. established a biomarker signature of twenty gut microbiome‐related metabolites, showing an AUC of 0.80 for CRC and 0.66 for CRA [19]. However, there were no common biomarkers between the two studies. In the current study, we first conducted two case–control studies to reduce the risk of false positivity, and then validated the model based on the same platform and protocols using data from another independent population, ensuring the validity and generalizability of our findings. The validated performance of our model for CRA versus NC (AUC = 0.89) is greater than those previously reported. The model also outperforms the current noninvasive tools clinically used for CRA diagnosis, such as the multi‐target fecal DNA test and FIT, which have reported sensitivities of only 42% [21] and 40% [8], respectively. Furthermore, we identified a panel of six metabolites for distinguishing CRC from CRA with AUC of 0.905 in the training set and 0.776 in the validation set. Only a few studies have focused on identifying biomarkers to distinguish CRC from CRA. One study reported that a panel of 13 gut microbiome‐related metabolites differentiated CRC from CRA with an AUC of 0.81 [19], while another study found that diacyl‐glycerophosphocholines C36:5 discriminated CRC from CRA with an AUC of 0.83 [22]. Neither of the studies validated their findings in an independent cohort.

One of the key findings from our study is that significant alterations in lipid metabolism were associated with colorectal carcinogenesis. Among 239 differential metabolites between CRC and NC, 170 metabolites could be classified into lipids and lipid‐like molecules and majority of them were downregulated in CRC patients. This pattern might reflect an increase in rate of lipid degradation as energy resources for the uncontrolled proliferation rate of malignant cells [23]. Referring to phospholipids, which have been extensively researched, as an illustration, a prior case–control study utilizing lipidomic approaches discovered that CRC patients had decreased levels of five phosphatidylcholines (PCs), four lyso‐phosphatidylcholine (LPCs) (also including LPC(14:0) and LPC(18:2)), and 11 phosphatidylethanolamine (PEs) in plasma as compared to healthy controls [24], lending support to our findings. In a two‐stage case–control study, serum levels of seven LPCs including LPC(15:0) were also found to be lower in CRC cases than in healthy individuals [25]. Moreover, a nested case–control study based on two prospective Shanghai cohorts revealed that lower levels of PC(22:6/18:0) and seven PEs were associated with an increased risk of CRC [26]. For adenoma, a case–control designed lipidomics study found that CRA patients had lower levels of five PCs in serum than healthy controls [27]. These epidemiological data indicate a potential role of certain phospholipids in CRC initiation, and further biological investigations are necessary to confirm the hypothesis.

The diagnostic potential of phospholipids and other lipid species has been previously proposed. For example, Li et al. found gradual decreases in plasma levels of major LPC species as CRC progressed, and certain choline‐containing phospholipids achieved 88% sensitivity and 80% specificity in distinguishing CRC from healthy controls [28]. Shen et al. identified LPC(18:2), LPC(18:3), PE(O‐36:3), PE(O‐38:3), ceramide(44:5), phosphatidylglycerol(34:0), and two sphingomyelins as promising diagnostic biomarkers for CRC, with AUC > 0.90 [24]. Similarly, in a plasma lipidomics study by Zhou et al., 12 lipids primarily consisting of PCs and FAs showed good diagnostic potential for CA (AUC ≥ 0.90) [27]. However, it remains unknown whether these biomarkers can perform well in external validation. To make metabolomic biomarkers clinically applicable, external validation is necessary. In this study, we utilized an untargeted metabolomics approach that encompassed a diverse range of metabolites, including numerous species of lipids. To derive optimal models for CRC and CRA, we selected metabolites of independent predictivity for CRC and CRA separately in a training set, and then externally validated the models. Our findings indicate that a panel of 10 metabolites, including two phospholipids (LPC(19:0) and LysoPI(18:0/0:0)), one FA derivative (methyl jasmonate), one glycerolipid (DGTS(2:0/20:1)), one lineolic acid derivative ((9S,10E,12Z,15Z)‐9‐hydroxy‐10,12,15‐octadecatrienoic acid), and one prenol lipid (carnosic acid) showed an AUC of 0.81 in the external validation for discriminating CRC from NC. The panel also showed a good discriminatory ability for early‐stage CRC with an AUC of 0.80. For CRA, a panel of seven metabolites, which incorporated five lipid species, was identified with a validated AUC of 0.89. Therefore, our results support that plasma metabolite biomarkers have potential to serve as a promising tool for the detection of CRC and CRA.

Apart from lipids, a previous case–control study reported that serum level of benzoic acid was downregulated in CRC patients compared to those without CRC, and this metabolite could be useful for CRC diagnosis (AUC = 0.89) [29]. Our model for distinguishing CRC from NC also included a benzoic acid derivative, namely, 3,4‐dihydroxybenzoic acid. This compound has been suggested to have an inhibitory effect against intestinal carcinogenesis [30]. Additionally, as far as we know, this study for the first time identified an organoheterocyclic compound (2‐hydroxyxanthone), an organic acid derivative (4‐methyl‐2‐oxovaleric acid), and an organic oxygen compound (Cer/AS(d14:2/13:1)) as promising biomarkers for CRC detection. In vitro and vivo studies suggest that hydroxyxanthones may serve as an anticancer agent against several cancers including CRC [31]. The roles of 4‐methyl‐2‐oxovaleric acid and Cer/AS (d14:2/13:1) in CRC remain to be determined.

A major strength of this study is based on a two‐stage design with discovery and replication phases involving three independent case–control studies, because it enabled the exclusion of metabolites with inconsistent associations with the case–control status. Additionally, machine learning approaches were employed to identify reliable biomarker panels. However, several limitations should be acknowledged as well. First, the retrospective design of the study limited our ability to establish causal associations. Future studies with prospective designs and biological experiments would be useful in explaining the associations observed in the current study. Second, metabolomics data from the untargeted approach were relative quantification. Efforts are required to determine absolute concentrations of the biomarkers before clinical translation. Finally, it remains undetermined whether the differential metabolites in plasma could reflect the status in colorectal tissues. In vivo and in vitro experiments are warranted to clarify the origin and biological mechanisms of the identified metabolites.

In conclusion, our study revealed obvious differences in plasma metabolic profiles of CRC, CRA, and NC, suggesting significant perturbations of energy metabolism in colorectal carcinogenesis. Specific metabolites, particularly lipid and lipid‐like molecules, showed good potential to improve the early detection of CRC. These results provide new clues on metabolic mechanisms underlying CRC and afford novel biomarkers for future clinical utility.

## 4 Methods and Materials

### 4.1 Study Design and Population

Three case–control studies were conducted involving 975 participants. The Nanjing study recruited 112 CRC patients, 57 CRA patients, and 112 individuals as NC from the Second Affiliated Hospital of Nanjing Medical University in Nanjing, China. The Guangzhou study included 107 CRC, 107 CRA, and 107 NC cases from the Sixth Affiliated Hospital of Sun Yat‐sen University in Guangzhou, China. The Kunming study enrolled 91 CRC, 115 CRA, and 109 NC cases from the First Affiliated Hospital of Kunming Medical University, Kunming, China. Demographic characteristics of participants are shown in Table S4.

**FIGURE 6:**
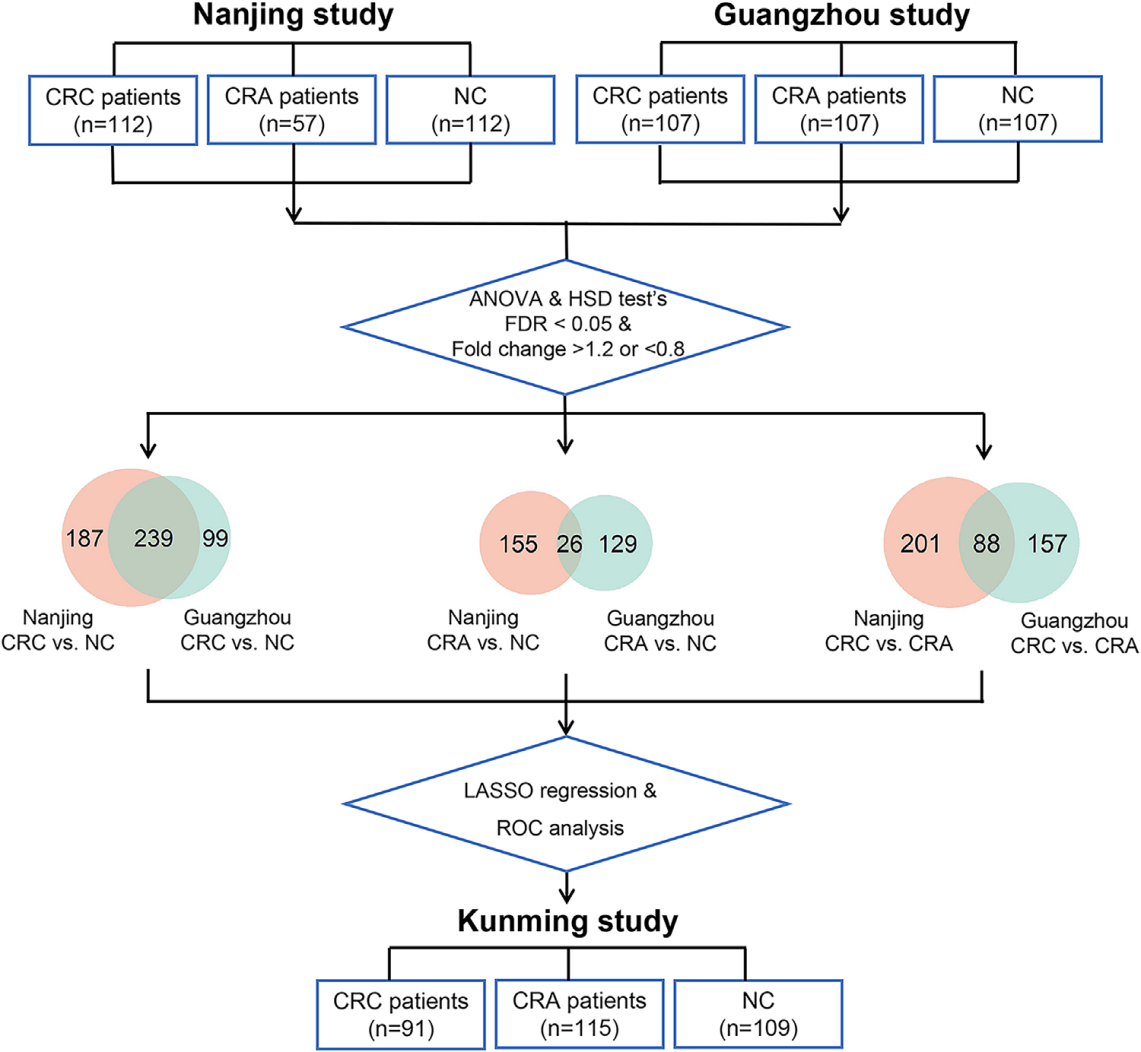
Schematic flow chart of the study. The case–control studies in Nanjing and Guangzhou (training set) were deigned to identify reproducible metabolic biomarkers of diagnostic potential in plasma using untargeted metabolomics. The case–control study in Kunming (validation set) was conducted to externally validate the diagnostic performance of selected metabolites. ANOVA, one‐way analysis of variance; CRA, colorectal adenoma; CRC, colorectal cancer; FDR, false discovery rate; HSD, honestly significant difference; LASSO, least absolute shrinkage and selection operator; NC, normal control; ROC, receiver operating characteristic.

All patients were newly diagnosed and confirmed by histology. Exclusion criteria included (1) receipt of any treatment before enrollment; (2) history of intestinal inflammation, infectious injury, or diarrhea; (3) history of surgery on colorectal or any malignant tumors; and (4) hereditary or familial CRC. The NC group was randomly selected from those with normal colonoscopy in the same hospital as the corresponding case group. In the Nanjing and Guangzhou studies, NC was 1:1 matched to CRC and CRA on age (±2 years) and sex. These two studies were designed to identify differential metabolites and train diagnostic models, while the Kunming study served as an external validation set (see flowchart in Figure 6).

To compare the performance of metabolites and CEA in aiding the diagnosis of CRC, we additionally recruited 92 CRC and 92 NC cases from the Zhejiang Cancer Hospital in Hangzhou, China. The recruitment and exclusion criteria were consistent with those employed in Nanjing and Guangzhou. Serum CEA concentrations, which were measured using an electrochemiluminescence immunoassay (CEA assay kit, Cobas e 801, Roche, Basel, Switzerland), were retrieved from medical records. To determine whether the plasma metabolites identified in this study were relatively specific diagnostic markers for CRC, we randomly recruited 55 patients with esophageal cancer, 55 with gastric cancer, 55 with liver cancer, 55 with breast cancer, and 55 NC from the biobank of the Zhejiang Cancer Hospital. To evaluate the change in plasma metabolites before and after treatment for CRC patients, we included an additional group of 25 CRC patients who provided blood samples both before and 5–7 days after surgery. To explore whether the metabolites with significant differences in plasma between CRC and NC were also differentially expressed in tumor and normal tissues, we included an additional group of 25 CRC patients who provided paired tumor and adjacent normal tissue samples.

### 4.2 Sample Collection and Metabolomic Profiling

Each participant provided approximately 5 mL of peripheral venous blood after fasting for a minimum of 8 h overnight. For patients with CRC and CRA, blood samples were drawn prior to any treatment. To determine the change in metabolites before and after treatment, a subset of CRC patients was asked to provide blood samples both before and 5–7 days after surgery. The blood samples were drawn by trained healthcare professionals using ethylenediaminetetraacetic acid anticoagulant tubes. Within 2 h of collection, the blood samples underwent rapid centrifugation to separate into plasma, red blood cells, and white blood cells, all of which were then stored at −80°C.

Additionally, tumor and adjacent normal tissues from CRC patients were obtained during surgery and subsequently flushed using phosphate‐buffered saline. After the operation, all tissue specimens were immediately snap‐frozen in liquid nitrogen.

The details of laboratory procedures have been outlined previously [32]. Briefly, an aliquot of plasma specimen (100 µL) was mixed with methanol containing isotope‐labeled internal standard (300 µL). Supernatants were separated by high‐speed centrifugation after vortexing, sonication, and incubation processes. To prepare the quality control sample, equal volumes of the supernatants from each of the samples were combined. High‐resolution and accurate profiling data were obtained using liquid chromatography‐mass spectrometry (LC‐MS) technique that comprised of UHPLC System (Vanquish, Thermo Fisher Scientific, Waltham, MA) coupled to a Orbitrap Exploris 120 mass spectrometer (Orbitrap MS, Thermo Fisher Scientific). Chromatography separation was achieved using UPLC HSS T3 column (2.1 mm × 100 mm, 1.8 µm) and a mobile phase consisting of 5 mmol/L ammonium acetate and 5 mmol/L acetic acid in water (A) and acetonitrile (B). The MS/MS spectra was acquired in a mode of information‐dependent acquisition, with electrospray ionization settings configured as follows: a sheath gas flow rate of 50 Arb, an aux gas flow rate of 15 Arb, a capillary temperature of 320°C, a full MS resolution of 60000, a MS/MS resolution of 15,000, a collision energy of 10/30/60 in normalized collision energy mode, and a spray voltage of 3.8 kV (positive) or −3.4 kV (negative).

### 4.3 Data Processing

The raw data of mass spectrometry were transformed into the mzXML format by the MS Convert software within ProteoWizard [33]. Peaks were detected, extracted, aligned, and integrated by an in‐house program (R package CAMERA). To ensure the data quality, peaks with a relative standard deviation greater than 0.3 in quality control samples and those with missing value (intensity = 0) in more than 50% of the samples were discarded. The LOESS method was used to normalize each peak for predicting and correcting intensity of the same metabolite across the real samples. [34] Metabolite was annotated by an in‐house MS2 database (Biotree, Shanghai, China). A total of 5736 metabolic features were detected in positive ionization mode, and 4626 features were detected in negative ionization mode. After concatenating the positive‐mode and negative‐mode features, 1117 endogenous metabolites were annotated (MS2 score > 0.3). For the current study, we included metabolites with a missingness rate less than 50% and imputed missing values with half of the minimum value of each metabolite across all samples. Finally, 891 named metabolites were analyzed.

### 4.4 Statistical Analysis

#### 4.4.1 Differential Metabolites Identification

To compare the differences in basic characteristics between CRC, CRA, and NC, continuous variables were analyzed with one‐way analysis of variance (ANOVA), while categorical variables were analyzed with chi‐square tests. To improve normality, metabolite data were natural‐log transformed. PLS‐DA was performed to visualize metabolomic discrimination between CRC, CRA, and NC. ANOVA with Tukey's HSD tests were used to identify differential metabolites. The false discovery rate (FDR) was calculated by the Benjamini–Hochberg procedure to correct for multiple comparisons. Metabolites with an FC > 1.2 or < 0.8 and *p*_FDR_ < 0.05 in both the Nanjing and Guangzhou studies were selected as candidate biomarkers. Heatmaps of differential metabolites were constructed.

#### 4.4.2 Pathway Analysis

In the enrichment analysis, Human Metabolome Database IDs were used to annotate metabolites and the pathway information was collected from Small Molecule Pathway Database (https://smpdb.ca/). Pathway enrichment analysis was conducted using MetaboAnalyst 5.0 [35]. We then conducted gene set enrichment analysis to obtain NES for the most significant pathway and depict the upregulation or downregulation of metabolites [36].

#### 4.4.3 Machine Learning

The LASSO algorithm with 1000 runs was used to further select the most reliable metabolites of diagnostic potential, requiring the presence in at least 700 runs [37]. To confirm the robustness of the LASSO selection, we applied the random forest method to select predictors as a sensitivity analysis. Differential metabolites with variable importance greater than three were chosen for model construction [38]. To evaluate the overall diagnostic performance of selected metabolites, AUC was calculated by LASSO and SVM with 1000 cross‐validation. The Youden Index was employed to identify the cut‐off point that maximized the combination of sensitivity and specificity. The DeLong test was used to evaluate the difference between the performance of metabolites and CEA in differentiating CRC from NC. All statistical analyses were conducted using R version 4.2.1.

## Author Contributions

**Jianv Huang**: formal analysis, methodology, writing – original draft. **Le Wang**: investigation, resources. **Xiang Zhang**: investigation, resources. **Xinyi Liu**: methodology. **Junyan Miao**: validation. **Yuefan Shen**: validation. **Chengqu Fu**: investigation. **Xianxiu Ge**: investigation. **Xue Wang**: investigation. **Jiancong Hu**: investigation. **Guanman Li**: investigation. **Yang Sun**: writing – review and editing. **Yinglei Miao**: writing – review and editing. **Juncheng Dai**: writing – review and editing. **Lingbin Du**: project administration, writing – review and editing. **Hongxia Ma**: methodology, writing – review and editing. **Guangfu Jin**: methodology, writing – review and editing. **Ni Li**: writing – review and editing. **Lin Miao**: resources. **Zhibin Hu**: resources, supervision. **Xiaosheng He**: funding acquisition, project administration, resources. **Jun Yu**: project administration, supervision, resources. **Hongbing Shen**: funding acquisition, resources, supervision. **Dong Hang**: conceptualization, funding acquisition, resources, supervision. All authors have read and approved the final manuscript.

## Ethics Statement

The study was approved by the Ethics Committees of Nanjing Medical University (approval number: 2020KY092), Sun Yat‐sen University (approval number: 2021ZSLYEC‐082), Kunming Medical University (approval number: 2022L95), and Zhejiang Cancer Hospital (approval number: IRB‐2023‐464). We obtained written informed consent from all the participants in this study.

## Conflicts of Interest

The authors declare no conflicts of interest.

## Supporting information



Supporting Information

## Data Availability

The raw data that support the findings of this study are openly available in EBI‐MetaboLights with accession number MTBLS12142 (www.ebi.ac.uk/metabolights/MTBLS12142).
