# Supplementary material for: Discovery and Validation of Novel Biomarkers for Colorectal Neoplasia Detection via Plasma Metabolomics
Source: MedComm (2020). 2025 Jun 6;6(6):e70201. doi: 10.1002/mco2.70201 (PMC12141919; doi:10.1002/mco2.70201)
Supplement: Supplementary file 1 — Supporting Information [file MCO2-6-e70201-s001.docx]

**Supplementary Materials**

**Discovery and validation of novel biomarkers for colorectal neoplasia detection via plasma metabolomics**

Jianv Huang^1,#^, Le Wang^2,#^, Xiang Zhang^3,#^, Xinyi Liu^1^, Junyan Miao^1^, Yuefan Shen^1^, Chengqu Fu^1^, Xianxiu Ge^1^, Xue Wang^2^, Jiancong Hu^5^, Guanman Li^5^, Yang Sun^6^, Yinglei Miao^6^, Juncheng Dai^1^, Lingbin Du^2^, Hongxia Ma^1^, Guangfu Jin^1^, Ni Li^7^, Lin Miao^4^, Zhibin Hu^1^, Xiaosheng He^5,^**^*^**, Jun Yu^3,^**^*^**, Hongbing Shen^1^, Dong Hang^1,^**^*^**

^1^ Department of Epidemiology, Jiangsu Key Lab of Cancer Biomarkers, Prevention and Treatment, Collaborative Innovation Center for Cancer Personalized Medicine, School of Public Health, Nanjing Medical University, Nanjing, China.

^2^ Zhejiang Provincial Office for Cancer Prevention and Control, Zhejiang Cancer Hospital, Institute of Basic Medicine and Cancer (IBMC), Chinese Academy of Sciences, Hangzhou, China.

^3^ Institute of Digestive Disease, Department of Medicine and Therapeutics, State Key Laboratory of Digestive Disease, Li Ka Shing Institute of Health Sciences, CUHK Shenzhen Research Institute, The Chinese University of Hong Kong, Hong Kong SAR, China.

^4^ Medical Centre for Digestive Diseases, the Second Affiliated Hospital of Nanjing Medical University, Nanjing, China.

^5^ Department of Colorectal Surgery, Guangdong Provincial Key Laboratory of Colorectal and Pelvic Floor Diseases, Guangdong Institute of Gastroenterology, the Sixth Affiliated Hospital of Sun Yat-sen University, Guangzhou, China.

^6^Department of Gastroenterology, The First Affiliated Hospital of Kunming Medical

University, Kunming, China.

^7^ Office of Cancer Screening, National Cancer Center/National Clinical Research Center for Cancer/Cancer Hospital, Chinese Academy of Medical Sciences and Peking Union Medical College, Beijing, China.

^#^ Co-first authors, contributed equally.

^*^ Co-corresponding authors

**Contents**

Figure S1…………………………………………………………………….…….....…………………………3

Figure S2…………………………………………………………………….…….....…………………………4

Figure S3…………………………………………………………………….…….....…………………………5

Figure S4…………………………………………………………………….……....…….……………………6

Figure S5…………………………………………………………………….…….....…………………………7

Figure S6…………………………………………………………………….…….....…………………………8

Figure S7…………………………………………………………………….…….....…………………………9

Figure S8…………………………………………………………………….…….....………………….…….10

Figure S9…………………………………………………………………….…….....………………….….…11

Figure S10…………………………………………………………………….…….....……………….….…..12

Figure S11…………………………………………………………………….…….....……………….….…..13

Figure S12…………………………………………………………………….…….....…………….…….…..14

Figure S13…………………………………………………………………….…….....…………….…….…..15

Figure S14…………………………………………………………………….…….....…………………..…..16

Table S1…………………………………………………………………….………...……………....….……17

Table S2………………………………………………………...………….…….....……...……………….…33

Table S3………………………………………………………...………….…….....……..………………..…34

Table S4………………………………………………………...………….…….....……..………………..…35


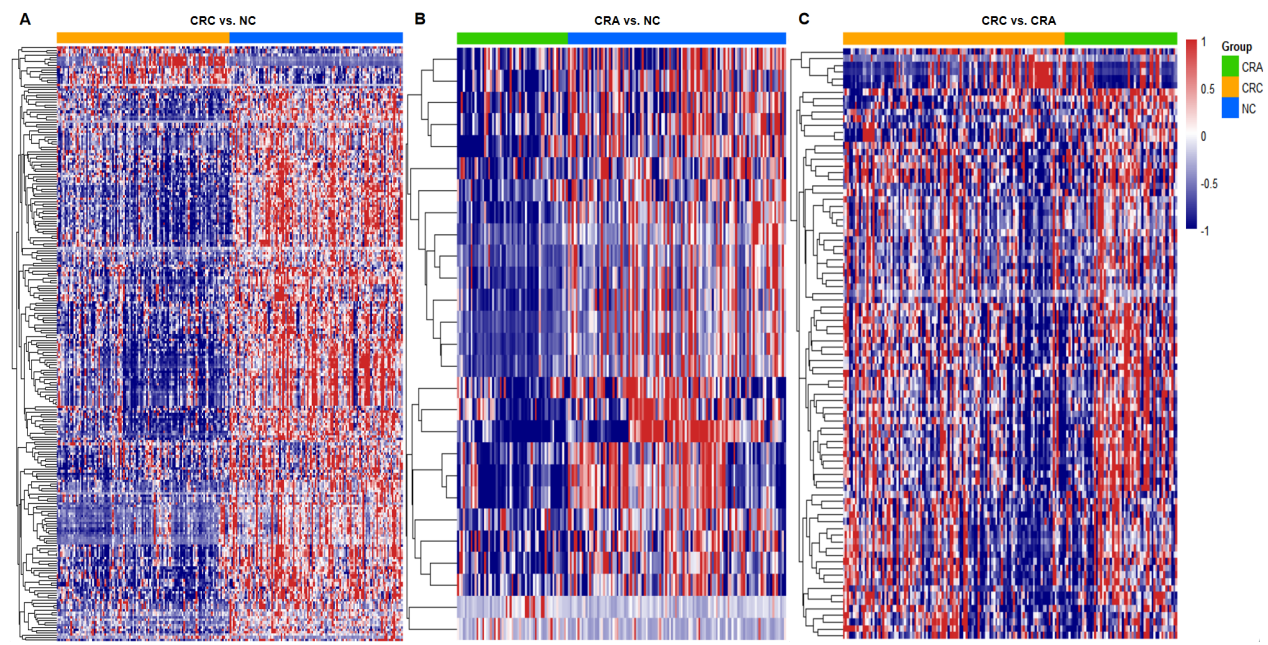


**Figure S1**. Heatmaps of differential metabolites identified in Nanjing. (A) CRC vs. NC. (B) CRA vs. NC. (C) CRC vs. CRA. Abbreviations: CRA, colorectal adenoma; CRC, colorectal cancer; NC, normal control.


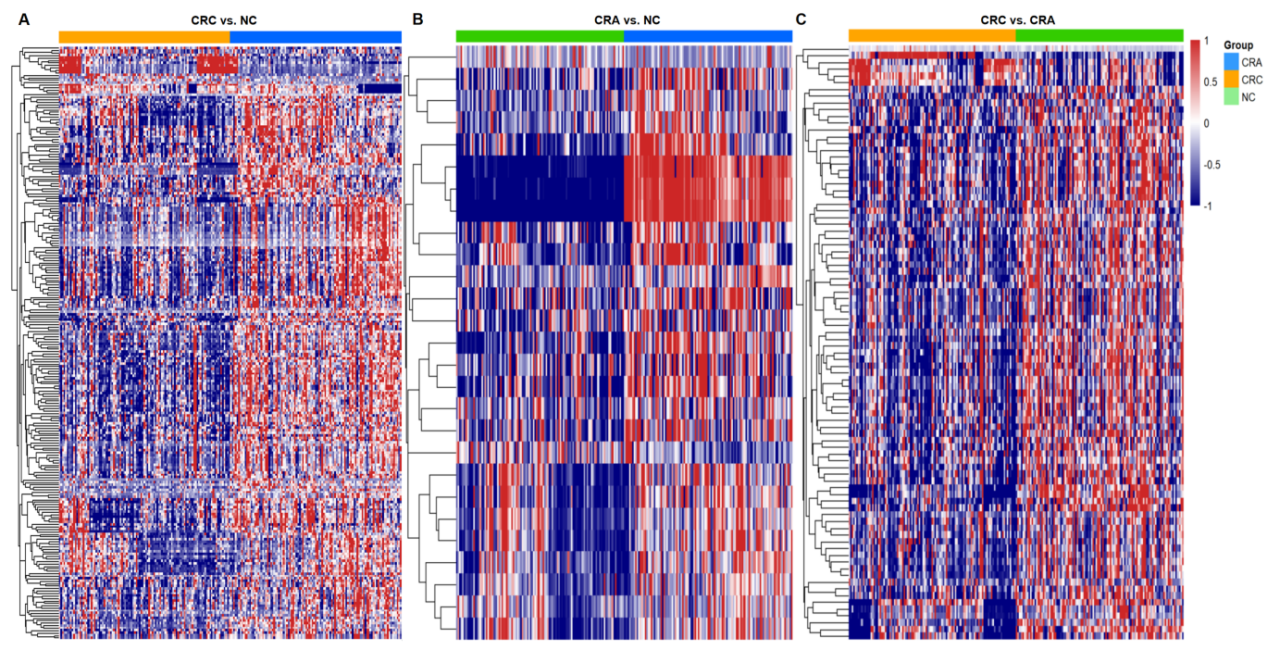


**Figure S2**. Heatmaps of differential metabolites identified in Guangzhou. (A) CRC vs. NC. (B) CRA vs. NC. (C) CRC vs. CRA. Abbreviations: CRA, colorectal adenoma; CRC, colorectal cancer; NC, normal control.


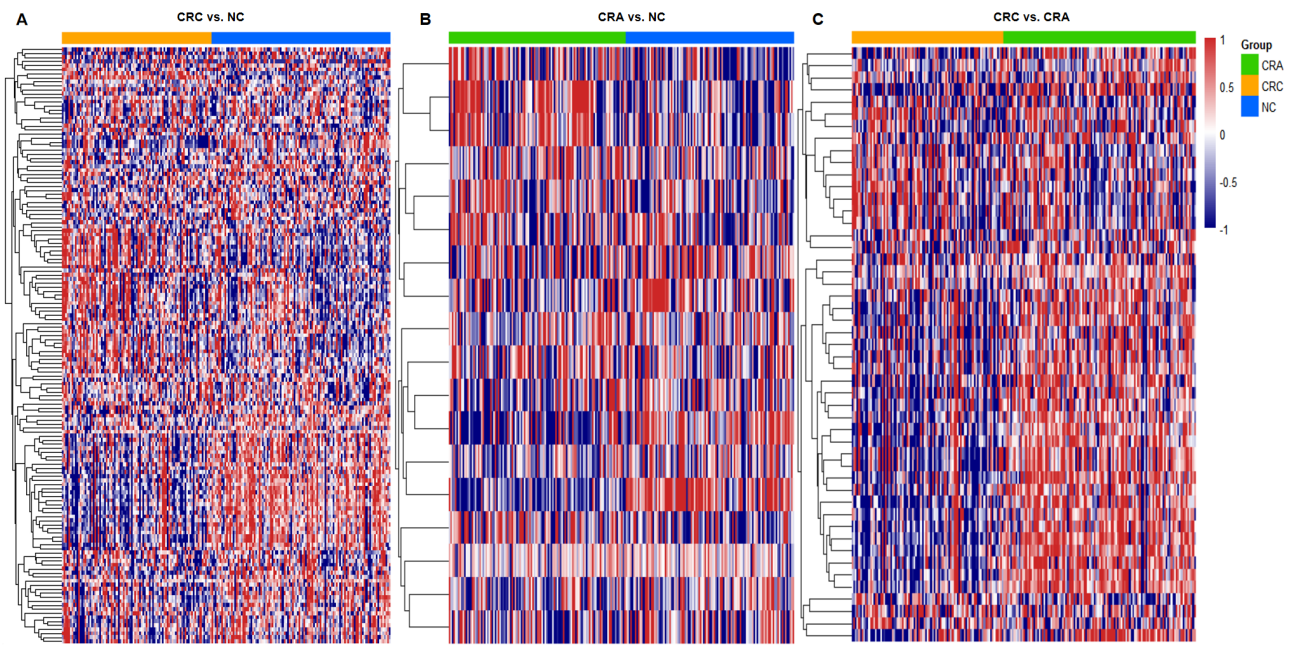


**Figure S3**. Heatmaps of differential metabolites identified in Kunming. (A) CRC vs. NC. (B) CRA vs. NC. (C) CRC vs. CRA. Abbreviations: CRA, colorectal adenoma; CRC, colorectal cancer; NC, normal control.


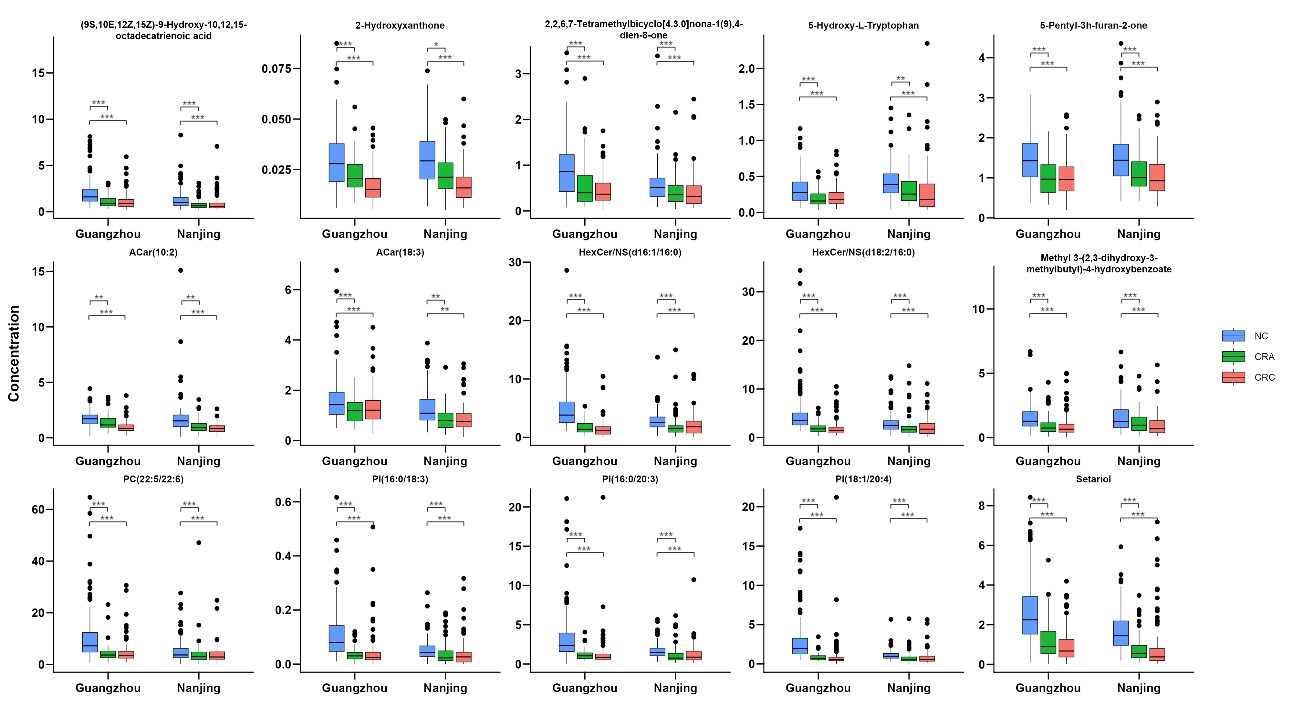


**Figure S4.** Box plots of 15 common differential metabolites observed in both CRC vs. NC and CRA vs. NC in the Guangzhou and Nanjing studies. Significance: ***FDR<0.001; **FDR<0.01; *FDR<0.05. Abbreviations: NC, normal control; CRA, colorectal adenoma; CRC, colorectal cancer.


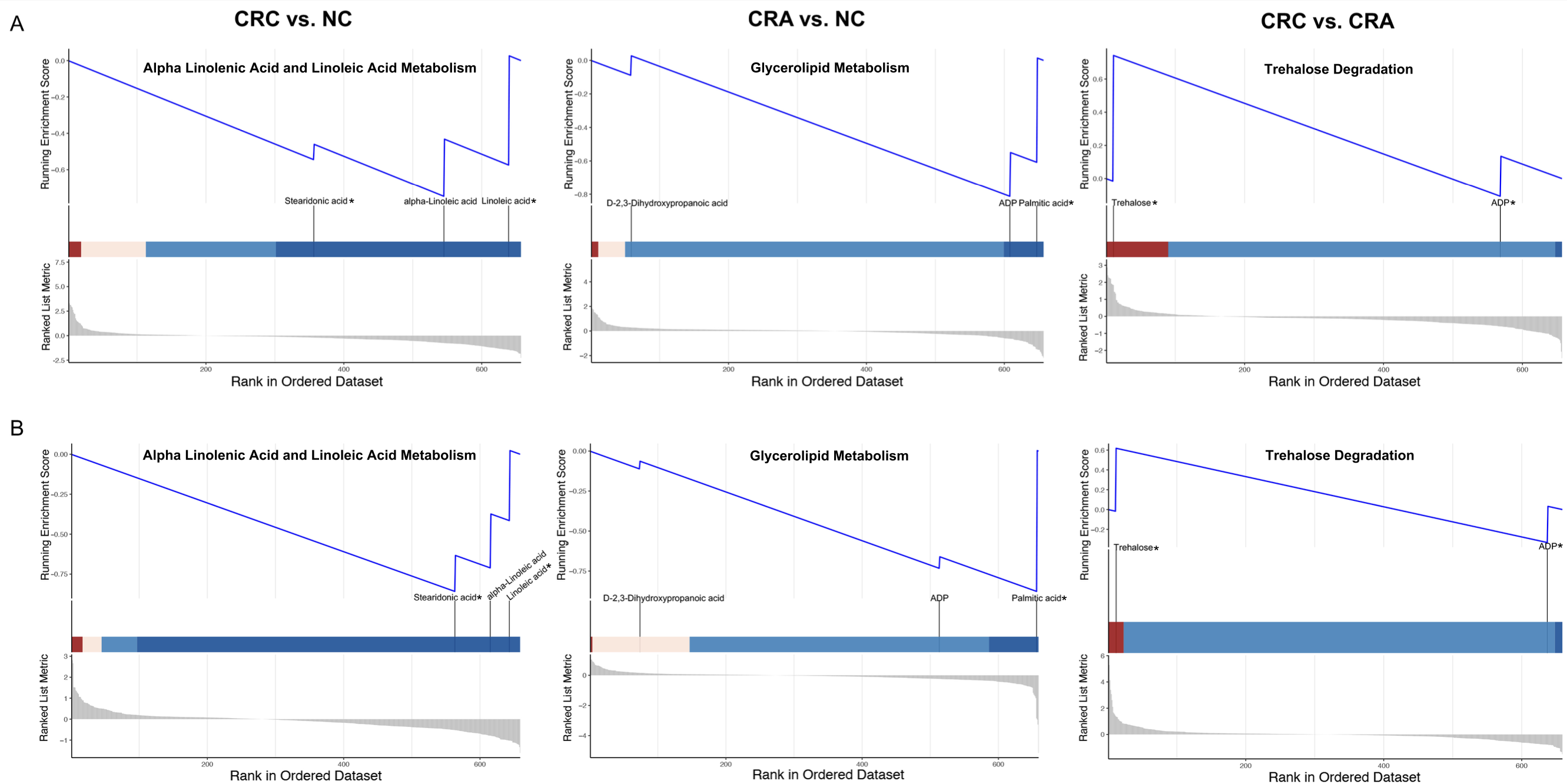


**Figure S5.** The gene set enrichment analysis for the most significant pathway. A) Nanjing; B) Guangzhou. An asterisk represents the differential metabolite also identified by ANOVA with Tukey’s HSD test. Abbreviations: ADP, adenosine diphosphate; CRA, colorectal adenoma; CRC, colorectal cancer; NC, normal control.


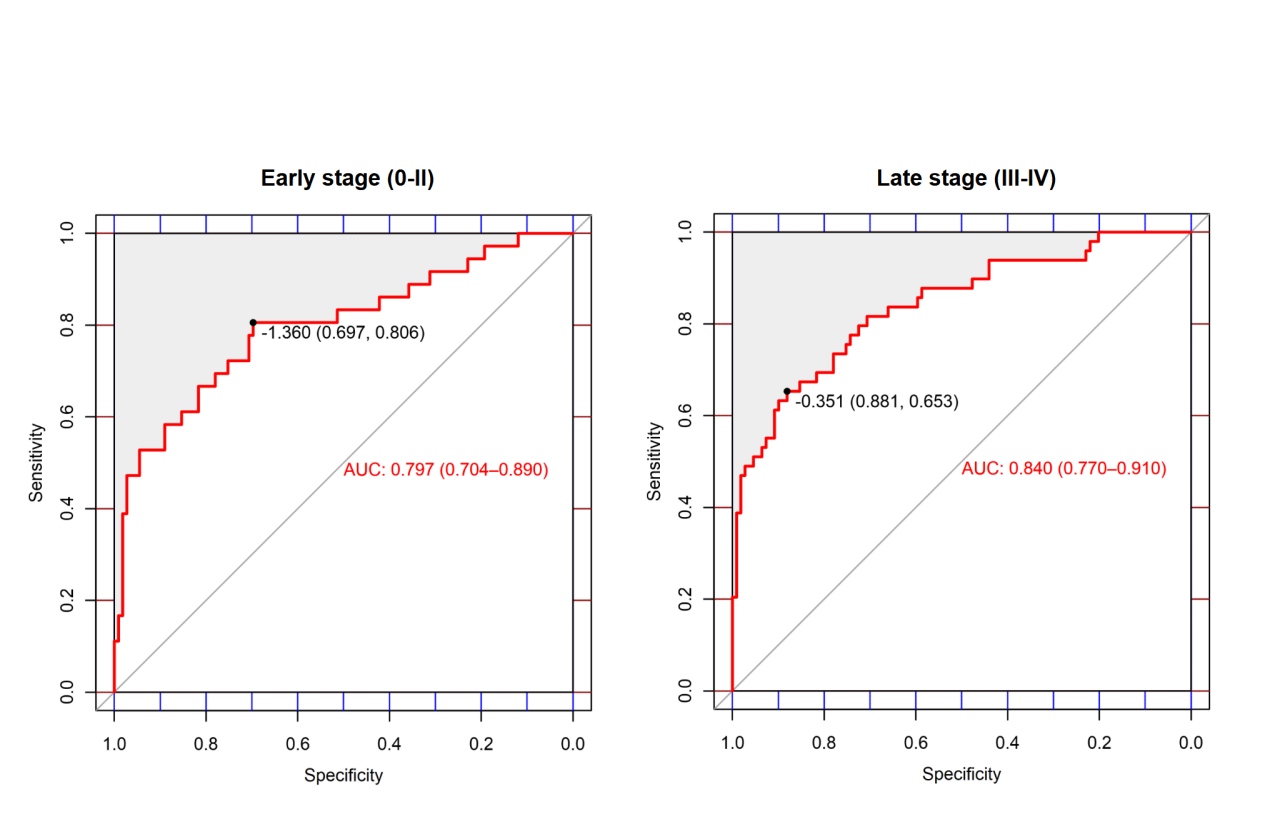


**Figure S6.** Receiver operator characteristic analysis of 10 metabolic biomarkers for discriminating early- (0-II) or late-stage (III-IV) colorectal cancer from normal control in the validation set. Abbreviations: AUC, area under the receiver operating characteristic curve.


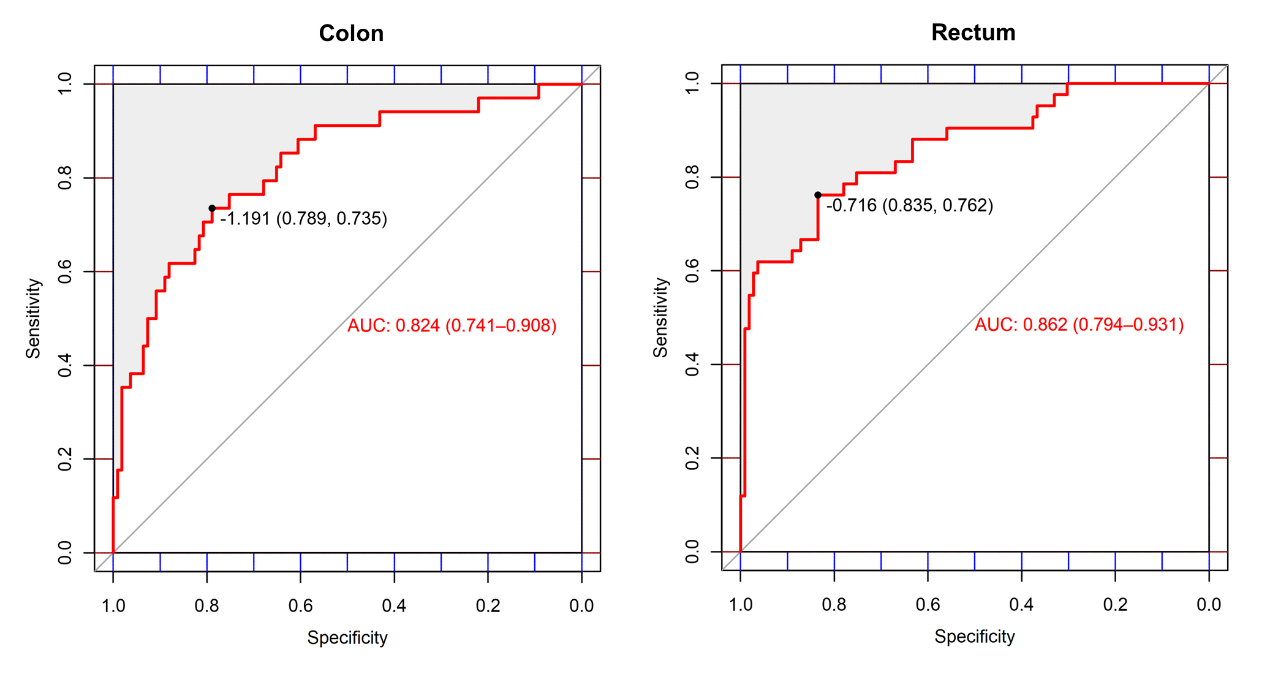


**Figure S7.** Receiver operator characteristic analysis of 10 metabolic biomarkers for discriminating colon or rectum cancer from normal control in the validation set. Abbreviations: AUC, area under the receiver operating characteristic curve.


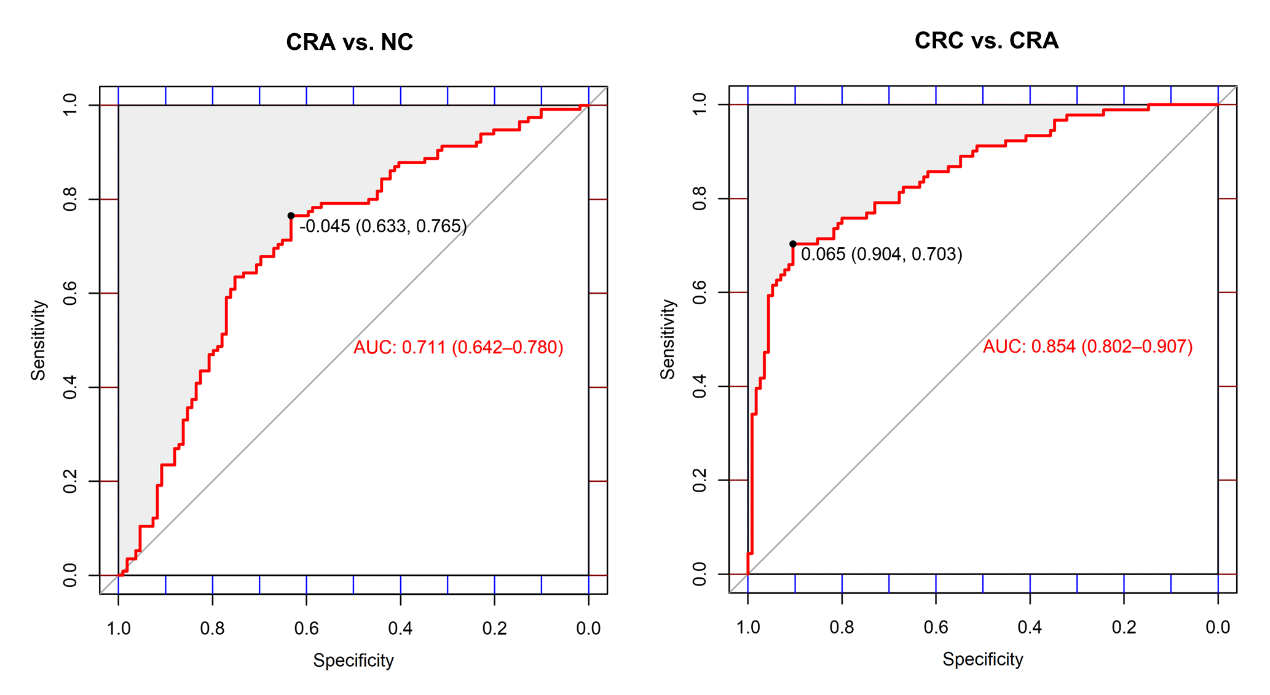


**Figure S8.** Receiver operator characteristic analysis of 10 metabolic biomarkers for discriminating CRA from NC and discriminating CRC from CRA in the validation set. Abbreviations: CRA, colorectal adenoma; NC, normal control; CRC, colorectal cancer. AUC, area under the receiver operating characteristic curve.


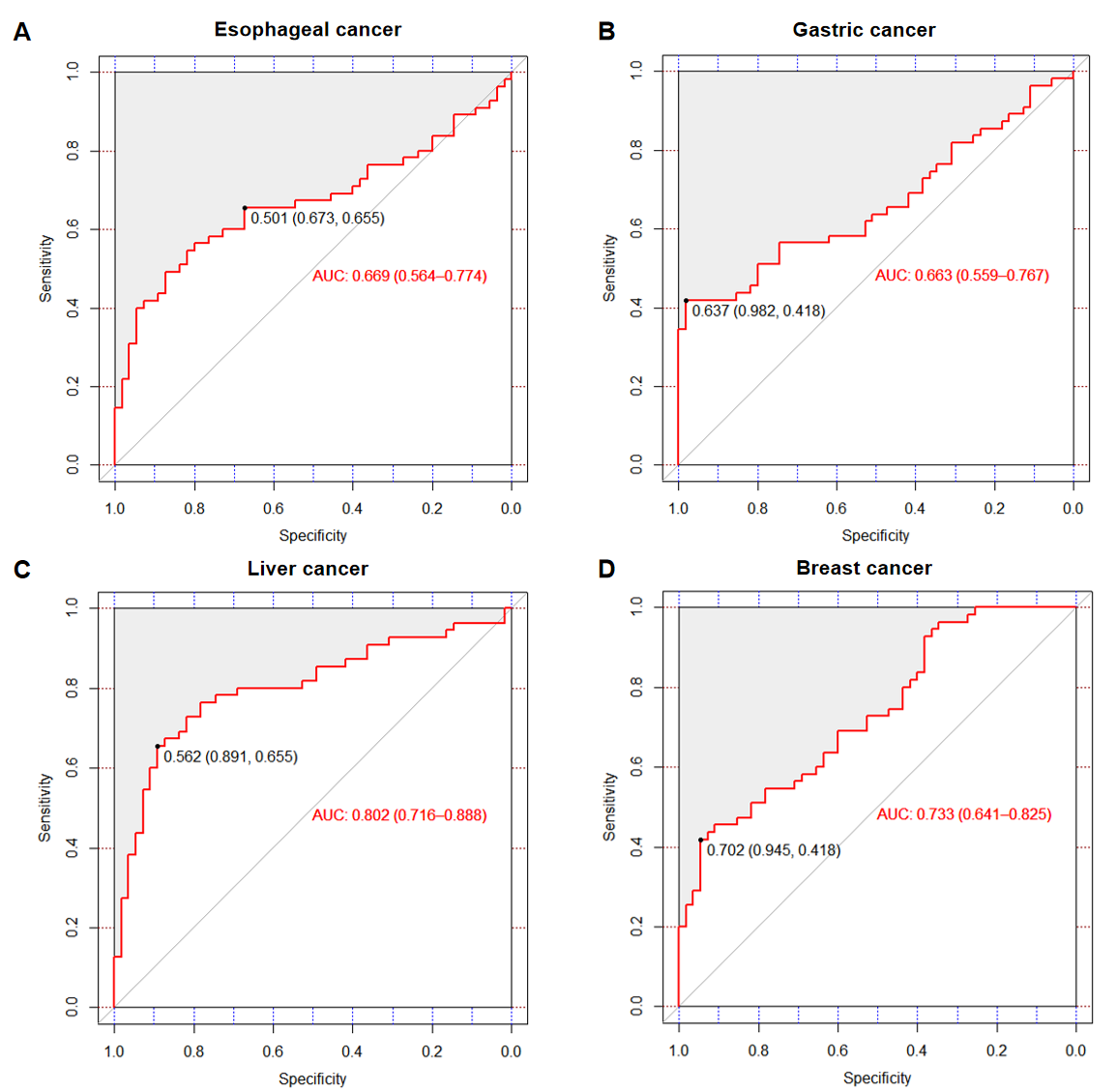


**Figure S9**. Receiver operating characteristic curves for plasma metabolites to discriminate esophageal cancer from NC (A), gastric cancer from NC (B), liver cancer from NC (C), and breast cancer from NC (D) in the Hangzhou study. Abbreviations: AUC, area under the receiver operating characteristic curve; NC, normal control.


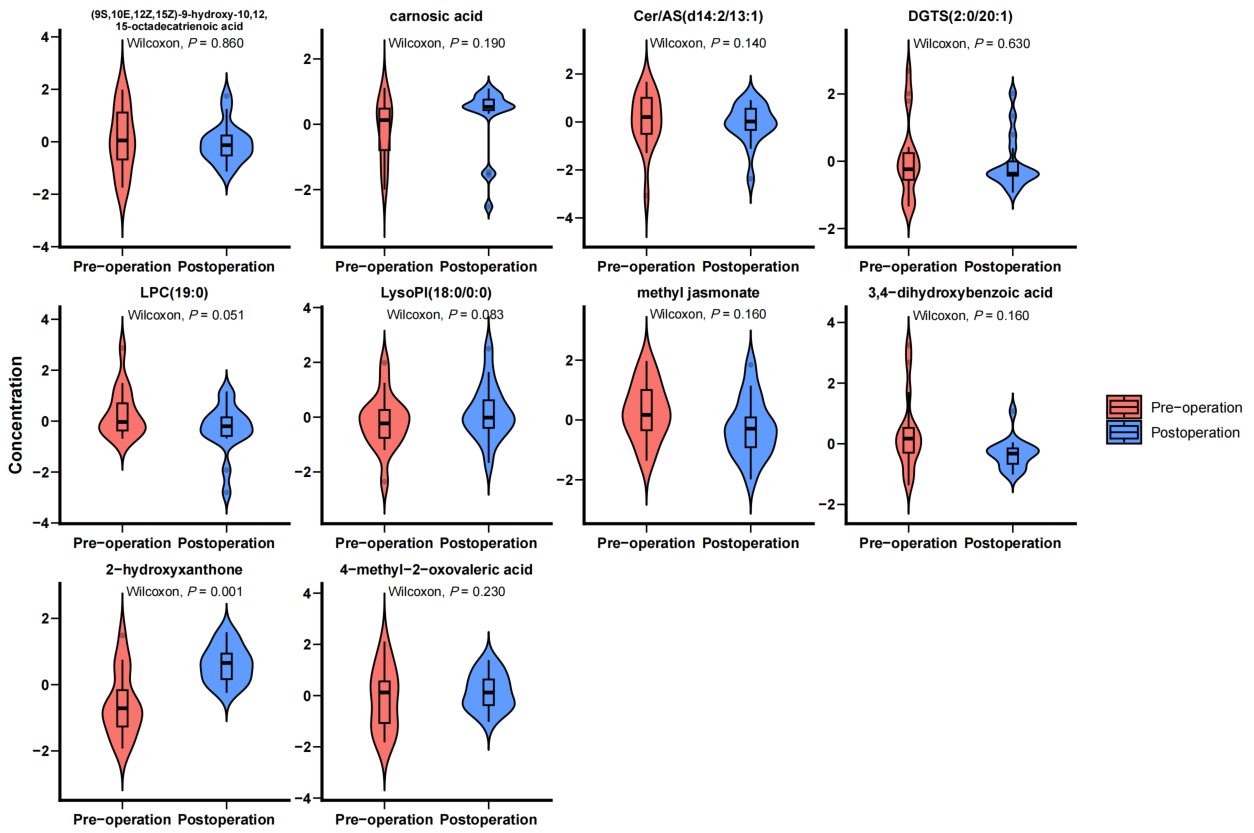


**Figure S10**. The levels of ten metabolites included in the metabolic panel for distinguishing CRC and NC were measured in plasma samples before and after surgery. Abbreviations: CRC, colorectal cancer; NC, normal control.


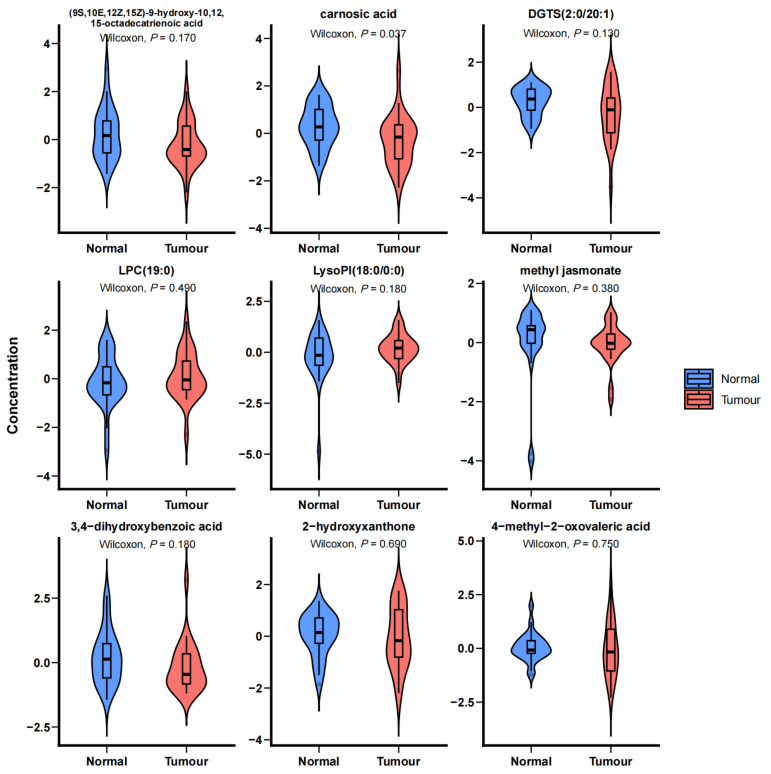


**Figure S11**. The levels of metabolites selected for distinguishing CRC and NC were measured in tumour and adjacent normal tissues. Abbreviations: CRC, colorectal cancer; NC, normal control.


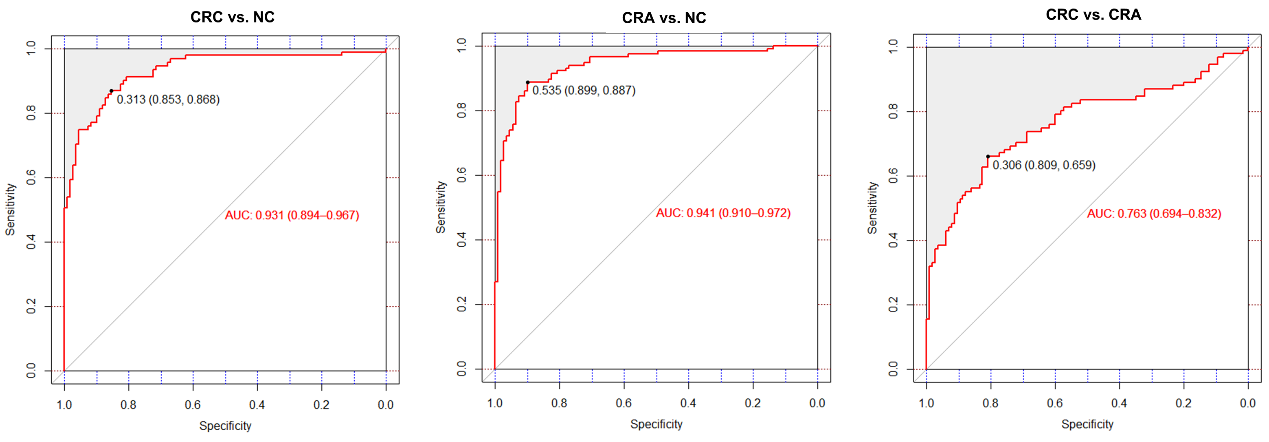


**Figure S12.** Receiver operator characteristic analysis of metabolic biomarkers based on support vector machine in the validation set. The models consisted of 10 metabolites for discriminating CRC from NC, 7 metabolites for discriminating CRA from NC, and 6 metabolites for discriminating CRC from CRA. Abbreviations: CRC, colorectal cancer; NC, normal control; CRA, colorectal adenoma; AUC, area under the receiver operating characteristic curve.


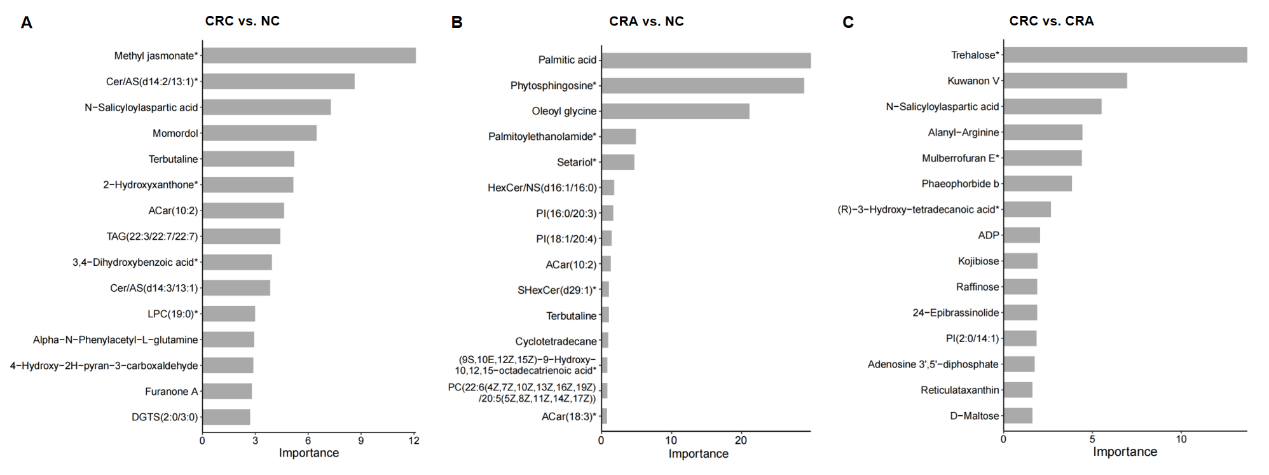


**Figure S13**. Plots of the random forest showing the metabolites with the top 15 VIP scores for distinguishing CRC from NC (A), CRA from NC (B), and CRC from CRA (C) in Guangzhou. The asterisk denotes the same metabolite in LASSO. Abbreviations: CRA, colorectal adenoma; CRC, colorectal cancer; LASSO, least absolute shrinkage and selection operator; NC, normal control, VIP, variable importance in projection.


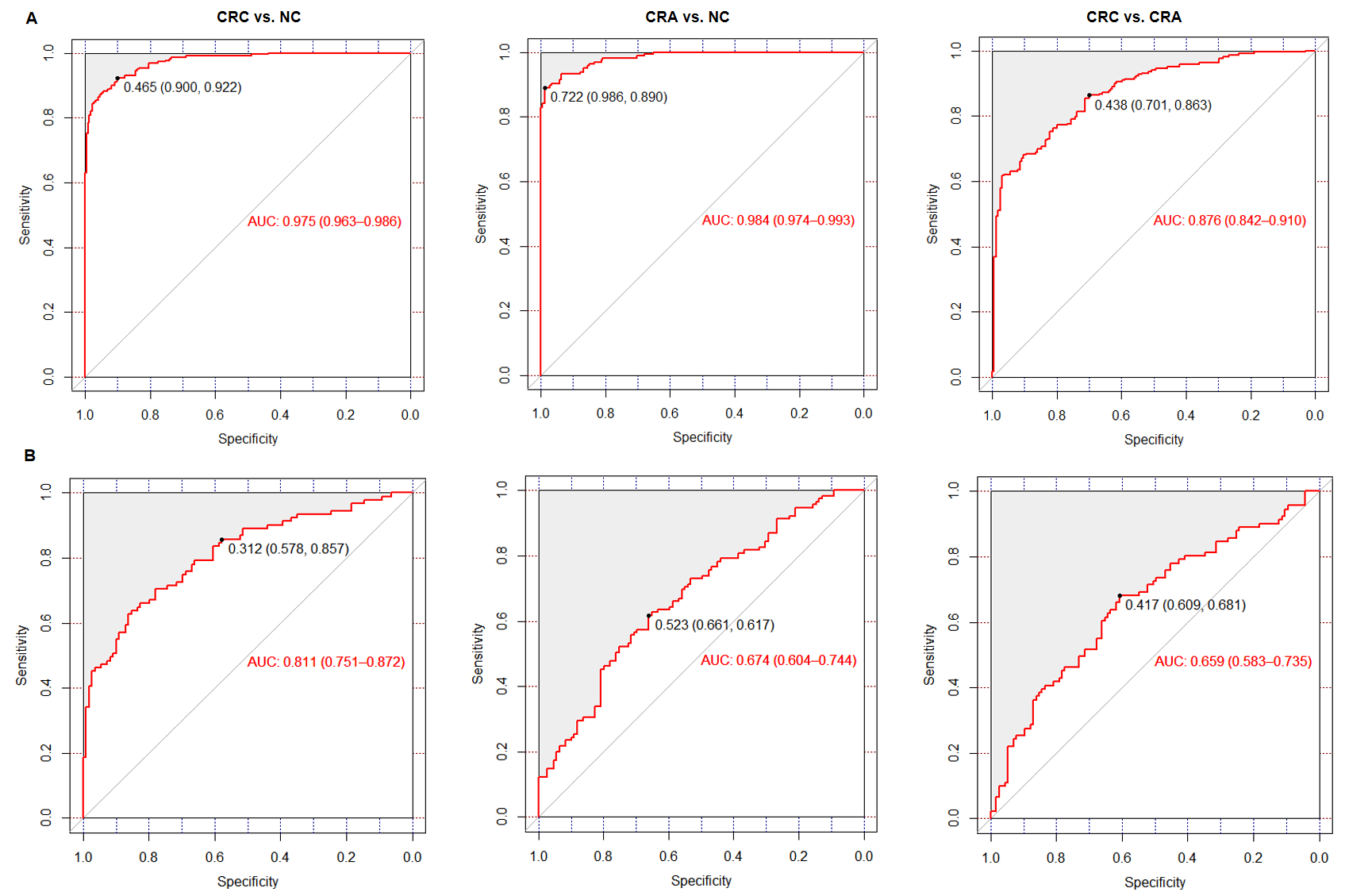


**Figure S14**. Receiver operating characteristic curves for plasma metabolites selected by the random forest in the training (A) and validation (B) sets. Abbreviations: CRA, colorectal adenoma; CRC, colorectal cancer; NC, normal control.

**Table S1.** Plasma differential metabolites between colorectal cancer, colorectal adenoma, and normal control in the Nanjing and Guangzhou studies

| **Metabolites** | **HMDB/LipidBlast** | **Super Class** | **Class** | **Nanjing study** | | **Guangzhou study** | | **Conditional logistic regression** | |
| --- | --- | --- | --- | --- | --- | --- | --- | --- | --- |
|  |  |  |  | **Fold change** | **FDR** † | **Fold change** | **FDR** † | OR (95% CI) ‡ | P **value** |
| **CRC vs. NC** |  |  |  |  |  |  |  |  |  |
| (10betaH,11xi)-11-Hydroxy-13-nor-6-eremophilen-8-one | HMDB0037605 | Lipids and lipid-like molecules | Prenol lipids | 0.379 | 2.28E-12 | 0.541 | 4.67E-11 | 0.15 (0.09, 0.25) | 2.20E-14 |
| (10E,12Z)-9-HODE | HMDB0062652 | Lipids and lipid-like molecules | Fatty acyls | 0.661 | 2.91E-05 | 0.715 | 4.25E-06 | 0.51 (0.40, 0.65) | 5.67E-08 |
| (3beta,5alpha,6beta,7alpha,22E,24R)-Ergosta-8,22-diene-3,5,6,7-tetrol | HMDB0032107 | Lipids and lipid-like molecules | Steroids and steroid derivatives | 0.348 | 2.28E-12 | 0.581 | 2.48E-10 | 0.17 (0.10, 0.27) | 1.61E-13 |
| (9E)-Valenciaxanthin | HMDB0036848 | Lipids and lipid-like molecules | Prenol lipids | 0.590 | 2.28E-12 | 0.732 | 1.02E-03 | 0.39 (0.29, 0.51) | 2.82E-11 |
| (9S,10E,12Z,15Z)-9-Hydroxy-10,12,15-octadecatrienoic acid | HMDB0031934 | Lipids and lipid-like molecules | Lineolic acids and derivatives | 0.531 | 4.39E-12 | 0.763 | 1.72E-05 | 0.41 (0.31, 0.54) | 6.28E-10 |
| (9S,10S)-9,10-dihydroxyoctadecanoate | HMDB0059633 | Lipids and lipid-like molecules | Fatty acyls | 0.510 | 2.36E-10 | 0.644 | 3.06E-06 | 0.43 (0.33, 0.56) | 3.09E-10 |
| (E)-2,6-Dimethyl-2,5-heptadienoic acid | HMDB0035135 | Lipids and lipid-like molecules | Fatty acyls | 0.774 | 1.53E-06 | 0.768 | 1.28E-06 | 0.46 (0.35, 0.59) | 1.79E-09 |
| (R)-3-Hydroxy-tetradecanoic acid | HMDB0010731 | Lipids and lipid-like molecules | Fatty acyls | 0.444 | 2.28E-12 | 0.621 | 1.34E-06 | 0.32 (0.24, 0.43) | 5.30E-14 |
| 12,13-DHOME | HMDB0004705 | Lipids and lipid-like molecules | Fatty acyls | 0.413 | 2.28E-12 | 0.510 | 2.39E-07 | 0.37 (0.28, 0.50) | 3.15E-11 |
| 12,13-EpOME | HMDB0004702 | Lipids and lipid-like molecules | Fatty acyls | 0.492 | 2.67E-12 | 0.571 | 1.28E-06 | 0.39 (0.30, 0.52) | 9.91E-11 |
| 12alpha-Hydroxy-13,18-dehydroparain | HMDB0039557 | Lipids and lipid-like molecules | Prenol lipids | 0.384 | 2.28E-12 | 0.704 | 4.24E-06 | 0.26 (0.18, 0.38) | 1.90E-13 |
| 12'-Apo-b-carotene-3,12'-diol | HMDB0036054 | Lipids and lipid-like molecules | Prenol lipids | 0.451 | 2.28E-12 | 0.566 | 2.40E-04 | 0.38 (0.28, 0.50) | 1.26E-11 |
| 12-Keto-leukotriene B4 | HMDB0004234 | Lipids and lipid-like molecules | Fatty acyls | 0.621 | 2.77E-10 | 0.580 | 3.85E-07 | 0.32 (0.22, 0.45) | 1.37E-10 |
| 16-Hydroxy hexadecanoic acid | HMDB0006294 | Lipids and lipid-like molecules | Fatty acyls | 0.743 | 2.62E-07 | 0.759 | 4.05E-05 | 0.49 (0.39, 0.63) | 1.16E-08 |
| 17-Hydroxyprogesterone | HMDB0000374 | Lipids and lipid-like molecules | Steroids and steroid derivatives | 0.581 | 2.28E-12 | 0.641 | 2.88E-08 | 0.25 (0.17, 0.37) | 1.10E-12 |
| 1-Acetoxy-2-hydroxy-16-heptadecyn-4-one | HMDB0031007 | Lipids and lipid-like molecules | Fatty acyls | 1.459 | 2.47E-04 | 1.413 | 3.58E-02 | 1.60 (1.28, 2.00) | 4.30E-05 |
| 1-Oleoylglycerophosphoinositol | HMDB0061693 | Lipids and lipid-like molecules | Glycerophospholipids | 0.597 | 2.28E-12 | 0.740 | 3.37E-06 | 0.28 (0.19, 0.39) | 1.87E-12 |
| 1-Palmitoylglycerophosphoinositol | HMDB0061695 | Lipids and lipid-like molecules | Glycerophospholipids | 0.559 | 2.28E-12 | 0.759 | 4.65E-06 | 0.27 (0.19, 0.38) | 1.23E-13 |
| 24,25-Dihydroxyvitamin D | HMDB0000430 | Lipids and lipid-like molecules | Steroids and steroid derivatives | 0.523 | 2.28E-12 | 0.722 | 6.69E-08 | 0.25 (0.17, 0.37) | 1.17E-12 |
| 24-Epibrassinolide | HMDB0041130 | Lipids and lipid-like molecules | Steroids and steroid derivatives | 0.429 | 2.28E-12 | 0.487 | 4.67E-11 | 0.14 (0.08, 0.24) | 3.58E-13 |
| 2-acetyl-1-alkyl-sn-glycero-3-phosphocholine | HMDB0062195 | Lipids and lipid-like molecules | Glycerophospholipids | 0.690 | 3.52E-09 | 0.787 | 1.47E-05 | 0.44 (0.34, 0.57) | 1.90E-09 |
| 3alpha-Acetomethoxy-11alpha-oxo-12-ursen-24-oic acid | HMDB0036673 | Lipids and lipid-like molecules | Prenol lipids | 0.623 | 4.46E-11 | 0.765 | 2.77E-05 | 0.41 (0.31, 0.55) | 1.64E-09 |
| 3-Oxocholic acid | HMDB0000502 | Lipids and lipid-like molecules | Steroids and steroid derivatives | 0.427 | 2.28E-12 | 0.463 | 4.67E-11 | 0.15 (0.09, 0.25) | 1.50E-13 |
| 5-Oxooctadecanoic acid | HMDB0034074 | Lipids and lipid-like molecules | Fatty acyls | 0.551 | 1.90E-10 | 0.604 | 1.27E-06 | 0.42 (0.32, 0.55) | 2.27E-10 |
| 5Z-Dodecenoic acid | HMDB0000529 | Lipids and lipid-like molecules | Fatty acyls | 0.432 | 2.28E-12 | 0.512 | 1.07E-05 | 0.34 (0.26, 0.46) | 2.91E-12 |
| 7-Ketocholesterol | HMDB0000501 | Lipids and lipid-like molecules | Steroids and steroid derivatives | 0.412 | 2.28E-12 | 0.569 | 4.67E-11 | 0.18 (0.11, 0.28) | 2.96E-13 |
| 9,10-Epoxyoctadecenoic acid | HMDB0004701 | Lipids and lipid-like molecules | Fatty acyls | 0.564 | 2.28E-12 | 0.622 | 2.65E-09 | 0.28 (0.20, 0.39) | 1.84E-13 |
| 9-Decenoic acid | HMDB0031003 | Lipids and lipid-like molecules | Fatty acyls | 0.432 | 2.28E-12 | 0.445 | 3.63E-09 | 0.29 (0.21, 0.40) | 1.02E-13 |
| 9-HODE | HMDB0010223 | Lipids and lipid-like molecules | Fatty acyls | 0.406 | 2.28E-12 | 0.497 | 5.47E-09 | 0.33 (0.24, 0.45) | 4.90E-12 |
| ACar(10:0) | LipidBlast000012 | Lipids and lipid-like molecules | Fatty acyls | 0.296 | 2.28E-12 | 0.462 | 4.98E-06 | 0.29 (0.21, 0.40) | 1.07E-13 |
| ACar(10:1) | LipidBlast000013 | Lipids and lipid-like molecules | Fatty acyls | 0.343 | 2.28E-12 | 0.487 | 2.58E-07 | 0.26 (0.19, 0.37) | 4.30E-14 |
| ACar(10:2) | LipidBlast000014 | Lipids and lipid-like molecules | Fatty acyls | 0.540 | 2.28E-12 | 0.501 | 3.08E-09 | 0.27 (0.20, 0.38) | 3.90E-14 |
| ACar(11:0) | LipidBlast000015 | Lipids and lipid-like molecules | Fatty acyls | 0.329 | 2.28E-12 | 0.700 | 8.39E-05 | 0.28 (0.20, 0.39) | 6.89E-13 |
| ACar(11:1) | LipidBlast000016 | Lipids and lipid-like molecules | Fatty acyls | 0.427 | 2.28E-12 | 0.633 | 2.58E-08 | 0.23 (0.16, 0.34) | 1.66E-13 |
| ACar(12:0) | LipidBlast000017 | Lipids and lipid-like molecules | Fatty acyls | 0.352 | 2.28E-12 | 0.505 | 4.51E-06 | 0.29 (0.21, 0.40) | 1.00E-13 |
| ACar(12:1) | LipidBlast000018 | Lipids and lipid-like molecules | Fatty acyls | 0.392 | 2.28E-12 | 0.677 | 1.21E-03 | 0.35 (0.26, 0.47) | 9.43E-13 |
| ACar(12:2) | LipidBlast000019 | Lipids and lipid-like molecules | Fatty acyls | 0.383 | 2.28E-12 | 0.575 | 2.93E-04 | 0.30 (0.22, 0.42) | 3.33E-13 |
| ACar(12:3) | LipidBlast000020 | Lipids and lipid-like molecules | Fatty acyls | 0.535 | 2.92E-11 | 0.530 | 2.48E-07 | 0.32 (0.23, 0.44) | 3.13E-12 |
| ACar(13:1) | LipidBlast000022 | Lipids and lipid-like molecules | Fatty acyls | 0.368 | 2.28E-12 | 0.738 | 6.38E-04 | 0.26 (0.19, 0.38) | 2.44E-13 |
| ACar(14:0) | LipidBlast000023 | Lipids and lipid-like molecules | Fatty acyls | 0.548 | 2.28E-12 | 0.662 | 1.26E-05 | 0.30 (0.22, 0.42) | 3.19E-13 |
| ACar(14:1) | LipidBlast000024 | Lipids and lipid-like molecules | Fatty acyls | 0.468 | 2.28E-12 | 0.612 | 1.09E-03 | 0.41 (0.32, 0.53) | 1.97E-11 |
| ACar(14:2) | LipidBlast000025 | Lipids and lipid-like molecules | Fatty acyls | 0.452 | 2.28E-12 | 0.572 | 7.39E-05 | 0.36 (0.27, 0.49) | 4.87E-12 |
| ACar(14:3) | LipidBlast000026 | Lipids and lipid-like molecules | Fatty acyls | 0.477 | 2.67E-12 | 0.480 | 1.94E-07 | 0.31 (0.23, 0.43) | 6.17E-13 |
| ACar(15:1) | LipidBlast000028 | Lipids and lipid-like molecules | Fatty acyls | 0.526 | 2.28E-12 | 0.753 | 4.72E-04 | 0.32 (0.23, 0.43) | 3.99E-13 |
| ACar(16:2) | LipidBlast000033 | Lipids and lipid-like molecules | Fatty acyls | 0.532 | 2.28E-12 | 0.588 | 7.18E-05 | 0.35 (0.26, 0.48) | 9.95E-12 |
| ACar(16:3) | LipidBlast000034 | Lipids and lipid-like molecules | Fatty acyls | 0.528 | 2.28E-12 | 0.650 | 3.95E-05 | 0.32 (0.23, 0.43) | 9.41E-13 |
| ACar(16:4) | LipidBlast000035 | Lipids and lipid-like molecules | Fatty acyls | 0.490 | 2.28E-12 | 0.621 | 1.47E-04 | 0.34 (0.25, 0.46) | 2.82E-12 |
| ACar(16:5) | LipidBlast000036 | Lipids and lipid-like molecules | Fatty acyls | 0.446 | 2.28E-12 | 0.551 | 4.24E-06 | 0.32 (0.24, 0.44) | 7.23E-13 |
| ACar(18:3) | LipidBlast000044 | Lipids and lipid-like molecules | Fatty acyls | 0.798 | 4.76E-03 | 0.673 | 7.76E-07 | 0.51 (0.40, 0.65) | 5.27E-08 |
| ACar(18:4) | LipidBlast000045 | Lipids and lipid-like molecules | Fatty acyls | 0.583 | 2.28E-12 | 0.756 | 1.35E-03 | 0.40 (0.30, 0.52) | 3.64E-11 |
| ACar(6:0) | LipidBlast000003 | Lipids and lipid-like molecules | Fatty acyls | 0.437 | 2.28E-12 | 0.575 | 1.59E-04 | 0.30 (0.22, 0.42) | 1.32E-12 |
| ACar(8:0) | LipidBlast000007 | Lipids and lipid-like molecules | Fatty acyls | 0.385 | 2.28E-12 | 0.446 | 4.14E-05 | 0.33 (0.24, 0.45) | 3.05E-12 |
| ACar(8:1) | LipidBlast000008 | Lipids and lipid-like molecules | Fatty acyls | 0.558 | 6.04E-12 | 0.590 | 1.12E-05 | 0.36 (0.26, 0.48) | 1.11E-11 |
| ACar(8:2) | LipidBlast000009 | Lipids and lipid-like molecules | Fatty acyls | 0.588 | 3.10E-08 | 0.587 | 2.51E-05 | 0.43 (0.33, 0.56) | 5.46E-10 |
| ACar(9:0) | LipidBlast000010 | Lipids and lipid-like molecules | Fatty acyls | 0.382 | 2.28E-12 | 0.669 | 8.31E-05 | 0.31 (0.22, 0.42) | 2.88E-13 |
| ACar(9:1) | LipidBlast000011 | Lipids and lipid-like molecules | Fatty acyls | 0.539 | 2.28E-12 | 0.650 | 3.37E-07 | 0.35 (0.26, 0.47) | 2.77E-12 |
| all-trans-Retinoic acid | HMDB0001852 | Lipids and lipid-like molecules | Prenol lipids | 1.218 | 1.13E-03 | 2.095 | 1.10E-03 | 1.93 (1.50, 2.49) | 3.86E-07 |
| But-2-enoic acid | HMDB0010720 | Lipids and lipid-like molecules | Fatty acyls | 1.282 | 1.59E-10 | 1.727 | 1.07E-07 | 2.19 (1.67, 2.89) | 2.20E-08 |
| Canthaxanthin | HMDB0003154 | Lipids and lipid-like molecules | Prenol lipids | 0.615 | 8.56E-07 | 0.693 | 9.30E-05 | 0.47 (0.36, 0.61) | 7.54E-09 |
| Carnosic acid | HMDB0002358 | Lipids and lipid-like molecules | Prenol lipids | 0.707 | 1.17E-07 | 0.629 | 5.69E-08 | 0.36 (0.27, 0.49) | 4.35E-11 |
| Cholestane-3,7,12,24,25-pentol | HMDB0002208 | Lipids and lipid-like molecules | Steroids and steroid derivatives | 0.338 | 2.28E-12 | 0.426 | 4.67E-11 | 0.15 (0.09, 0.25) | 2.84E-13 |
| Cholesterol sulfate | HMDB0000653 | Lipids and lipid-like molecules | Steroids and steroid derivatives | 0.447 | 2.28E-12 | 0.794 | 5.51E-04 | 0.27 (0.19, 0.39) | 3.93E-13 |
| Cholic acid | HMDB0000619 | Lipids and lipid-like molecules | Steroids and steroid derivatives | 0.526 | 2.28E-12 | 0.573 | 4.67E-11 | 0.26 (0.18, 0.37) | 9.70E-14 |
| Decanoylcarnitine | HMDB0000651 | Lipids and lipid-like molecules | Fatty acyls | 0.671 | 2.28E-12 | 0.775 | 1.64E-05 | 0.31 (0.22, 0.43) | 9.24E-12 |
| DG(18:3(9Z,12Z,15Z)/15:0/0:0) | HMDB0007300 | Lipids and lipid-like molecules | Glycerolipids | 0.465 | 2.28E-12 | 0.599 | 2.07E-07 | 0.37 (0.28, 0.49) | 1.10E-11 |
| DG(18:4(6Z,9Z,12Z,15Z)/15:0/0:0) | HMDB0007329 | Lipids and lipid-like molecules | Glycerolipids | 0.492 | 5.17E-10 | 0.628 | 2.52E-05 | 0.48 (0.37, 0.61) | 2.91E-09 |
| DGTS(2:0/16:3) | Not available | Lipids and lipid-like molecules | Glycerolipids | 0.642 | 3.02E-09 | 0.784 | 2.45E-03 | 0.40 (0.30, 0.54) | 5.70E-10 |
| DGTS(2:0/17:2) | Not available | Lipids and lipid-like molecules | Glycerolipids | 0.624 | 2.28E-12 | 0.771 | 4.50E-09 | 0.28 (0.20, 0.40) | 2.37E-13 |
| DGTS(2:0/20:1) | Not available | Lipids and lipid-like molecules | Glycerolipids | 0.252 | 2.28E-12 | 0.468 | 3.09E-07 | 0.25 (0.17, 0.37) | 1.73E-12 |
| DGTS(2:0/20:3) | Not available | Lipids and lipid-like molecules | Glycerolipids | 0.324 | 1.23E-10 | 0.627 | 2.27E-03 | 0.43 (0.33, 0.57) | 1.91E-09 |
| DGTS(2:0/3:0) | Not available | Lipids and lipid-like molecules | Glycerolipids | 2.136 | 3.60E-07 | 2.154 | 1.16E-07 | 2.23 (1.73, 2.88) | 7.07E-10 |
| Dolichosterone | HMDB0034336 | Lipids and lipid-like molecules | Steroids and steroid derivatives | 0.379 | 2.28E-12 | 0.461 | 4.67E-11 | 0.11 (0.06, 0.20) | 1.07E-13 |
| Eremopetasidione | HMDB0040778 | Lipids and lipid-like molecules | Prenol lipids | 0.762 | 4.55E-05 | 0.720 | 5.98E-08 | 0.41 (0.31, 0.54) | 1.83E-10 |
| Esculentic acid (Diplazium) | HMDB0035782 | Lipids and lipid-like molecules | Prenol lipids | 0.550 | 2.28E-12 | 0.600 | 2.21E-06 | 0.36 (0.27, 0.48) | 5.87E-12 |
| Ethyl dodecanoate | HMDB0033788 | Lipids and lipid-like molecules | Fatty acyls | 0.598 | 2.28E-12 | 0.700 | 6.29E-05 | 0.36 (0.27, 0.49) | 1.01E-11 |
| Ethyl menthane carboxamide | HMDB0037834 | Lipids and lipid-like molecules | Prenol lipids | 0.683 | 5.43E-05 | 0.733 | 7.43E-03 | 0.57 (0.46, 0.72) | 1.23E-06 |
| FA(16:2) | LipidBlast417785 | Lipids and lipid-like molecules | Fatty acyls | 0.631 | 4.23E-12 | 0.686 | 4.29E-05 | 0.38 (0.28, 0.51) | 2.99E-10 |
| FA(16:3) | LipidBlast417786 | Lipids and lipid-like molecules | Fatty acyls | 0.618 | 4.24E-12 | 0.687 | 3.83E-04 | 0.42 (0.32, 0.56) | 1.58E-09 |
| FA(16:4) | LipidBlast417787 | Lipids and lipid-like molecules | Fatty acyls | 0.484 | 2.28E-12 | 0.610 | 1.12E-05 | 0.37 (0.28, 0.49) | 1.97E-12 |
| FA(17:0) | HMDB0002259 | Lipids and lipid-like molecules | Fatty acyls | 0.713 | 2.74E-06 | 0.790 | 1.35E-02 | 0.55 (0.43, 0.70) | 1.65E-06 |
| FA(19:0) | LipidBlast417799 | Lipids and lipid-like molecules | Fatty acyls | 0.772 | 3.51E-06 | 0.696 | 7.59E-07 | 0.42 (0.32, 0.57) | 9.68E-09 |
| FA(19:4) | LipidBlast417803 | Lipids and lipid-like molecules | Fatty acyls | 0.615 | 5.05E-09 | 0.667 | 5.07E-04 | 0.48 (0.37, 0.62) | 2.27E-08 |
| FA(20:0) | HMDB0002212 | Lipids and lipid-like molecules | Fatty acyls | 0.762 | 5.01E-03 | 0.602 | 4.24E-06 | 0.51 (0.40, 0.66) | 2.76E-07 |
| FA(20:4) | LipidBlast417809 | Lipids and lipid-like molecules | Fatty acyls | 0.708 | 2.20E-11 | 0.786 | 8.91E-04 | 0.41 (0.30, 0.55) | 3.46E-09 |
| FA(21:3) | LipidBlast417815 | Lipids and lipid-like molecules | Fatty acyls | 0.434 | 2.28E-12 | 0.550 | 4.67E-11 | 0.27 (0.19, 0.38) | 1.10E-13 |
| FA(22:5) | LipidBlast417823 | Lipids and lipid-like molecules | Fatty acyls | 0.723 | 9.52E-06 | 0.759 | 2.42E-03 | 0.51 (0.39, 0.66) | 1.80E-07 |
| FA(22:6) | LipidBlast417824 | Lipids and lipid-like molecules | Fatty acyls | 0.654 | 2.28E-12 | 0.732 | 7.81E-05 | 0.38 (0.28, 0.50) | 4.82E-11 |
| FAHFA(16:0/3:0) | Not available | Lipids and lipid-like molecules | Fatty acyls | 0.782 | 1.72E-07 | 0.697 | 1.09E-05 | 0.45 (0.34, 0.60) | 4.19E-08 |
| FAHFA(18:1/22:2) | Not available | Lipids and lipid-like molecules | Fatty acyls | 0.777 | 4.11E-02 | 0.753 | 3.48E-04 | 0.60 (0.47, 0.76) | 2.77E-05 |
| FAHFA(2:0/22:2) | Not available | Lipids and lipid-like molecules | Fatty acyls | 0.563 | 2.28E-12 | 0.643 | 5.58E-11 | 0.28 (0.20, 0.39) | 9.60E-14 |
| Furanofukinin | HMDB0036640 | Lipids and lipid-like molecules | Prenol lipids | 0.331 | 2.28E-12 | 0.508 | 5.81E-11 | 0.23 (0.16, 0.34) | 3.50E-14 |
| Ganoderiol H | HMDB0037783 | Lipids and lipid-like molecules | Prenol lipids | 0.371 | 2.28E-12 | 0.503 | 4.81E-11 | 0.24 (0.17, 0.35) | 1.57E-13 |
| Geranic acid | HMDB0036103 | Lipids and lipid-like molecules | Prenol lipids | 0.760 | 3.87E-04 | 0.491 | 9.53E-07 | 0.41 (0.30, 0.55) | 1.03E-08 |
| Glycerol triundecanoate | HMDB0031089 | Lipids and lipid-like molecules | Glycerolipids | 0.570 | 2.44E-10 | 0.660 | 2.94E-06 | 0.40 (0.30, 0.53) | 2.11E-10 |
| Goshuyic acid | HMDB0000560 | Lipids and lipid-like molecules | Fatty acyls | 0.413 | 5.88E-12 | 0.615 | 3.68E-06 | 0.39 (0.29, 0.52) | 6.55E-11 |
| HexCer/NS(d14:1/12:1) | Not available | Lipids and lipid-like molecules | Fatty acyls | 0.740 | 4.06E-05 | 0.769 | 1.29E-04 | 0.46 (0.34, 0.62) | 2.95E-07 |
| HexCer/NS(d14:1/16:2) | Not available | Lipids and lipid-like molecules | Fatty acyls | 0.795 | 1.47E-03 | 0.743 | 3.83E-04 | 0.54 (0.41, 0.71) | 7.03E-06 |
| HexCer/NS(d14:2/16:2) | Not available | Lipids and lipid-like molecules | Fatty acyls | 0.616 | 1.99E-07 | 0.454 | 3.63E-09 | 0.36 (0.27, 0.50) | 2.51E-10 |
| HexCer/NS(d14:3/14:1) | Not available | Lipids and lipid-like molecules | Fatty acyls | 0.543 | 3.73E-11 | 0.643 | 8.53E-06 | 0.35 (0.26, 0.49) | 2.89E-10 |
| HexCer/NS(d16:1/16:0) | Not available | Lipids and lipid-like molecules | Fatty acyls | 0.290 | 2.28E-12 | 0.741 | 4.32E-05 | 0.26 (0.18, 0.38) | 6.93E-13 |
| HexCer/NS(d18:2/16:0) | Not available | Lipids and lipid-like molecules | Fatty acyls | 0.370 | 2.28E-12 | 0.742 | 2.12E-04 | 0.34 (0.25, 0.46) | 1.40E-12 |
| Hovenidulcigenin B | HMDB0041547 | Lipids and lipid-like molecules | Prenol lipids | 0.630 | 2.28E-12 | 0.774 | 1.53E-06 | 0.32 (0.24, 0.44) | 1.97E-12 |
| Hyodeoxycholic acid | HMDB0000733 | Lipids and lipid-like molecules | Steroids and steroid derivatives | 0.573 | 2.28E-12 | 0.764 | 1.40E-07 | 0.26 (0.18, 0.38) | 5.08E-12 |
| Isothankunic acid | HMDB0040772 | Lipids and lipid-like molecules | Prenol lipids | 0.504 | 2.28E-12 | 0.704 | 1.22E-07 | 0.20 (0.13, 0.31) | 2.11E-13 |
| Kojibiose | HMDB0011742 | Lipids and lipid-like molecules | Fatty acyls | 8.674 | 2.28E-12 | 1.252 | 3.02E-03 | 2.63 (1.96, 3.54) | 1.60E-10 |
| Leukotriene B4 | HMDB0001085 | Lipids and lipid-like molecules | Fatty acyls | 0.693 | 1.03E-08 | 0.716 | 3.08E-07 | 0.37 (0.28, 0.50) | 2.35E-11 |
| Linoleic acid | HMDB0000673 | Lipids and lipid-like molecules | Fatty acyls | 0.368 | 2.28E-12 | 0.491 | 4.67E-11 | 0.22 (0.15, 0.33) | 1.10E-13 |
| Lithocholyltaurine | HMDB0000722 | Lipids and lipid-like molecules | Steroids and steroid derivatives | 0.582 | 2.28E-12 | 0.743 | 1.09E-05 | 0.28 (0.20, 0.39) | 8.31E-13 |
| LPC(14:0) | HMDB0010379/LipidBlast454310 | Lipids and lipid-like molecules | Glycerophospholipids | 0.557 | 2.28E-12 | 0.667 | 1.09E-07 | 0.33 (0.24, 0.45) | 7.36E-12 |
| LPC(15:0) | HMDB0010381/LipidBlast454312 | Lipids and lipid-like molecules | Glycerophospholipids | 0.668 | 2.28E-12 | 0.747 | 3.65E-07 | 0.34 (0.24, 0.47) | 4.12E-11 |
| LPC(17:2) | LipidBlast454321 | Lipids and lipid-like molecules | Glycerophospholipids | 0.688 | 3.01E-08 | 0.670 | 1.15E-07 | 0.42 (0.32, 0.55) | 5.08E-10 |
| LPC(18:2) | HMDB0010386/LipidBlast454324 | Lipids and lipid-like molecules | Glycerophospholipids | 0.733 | 1.48E-03 | 0.712 | 5.90E-06 | 0.57 (0.45, 0.72) | 2.16E-06 |
| LPC(19:0) | LipidBlast454328 | Lipids and lipid-like molecules | Glycerophospholipids | 0.569 | 2.28E-12 | 0.654 | 6.78E-10 | 0.27 (0.19, 0.39) | 4.18E-13 |
| LPC(20:1) | HMDB0010391/LipidBlast454332 | Lipids and lipid-like molecules | Glycerophospholipids | 0.617 | 2.28E-12 | 0.794 | 4.84E-06 | 0.33 (0.24, 0.45) | 1.68E-11 |
| LPE(17:0) | LipidBlast454380 | Lipids and lipid-like molecules | Glycerophospholipids | 0.734 | 2.41E-06 | 0.791 | 1.79E-04 | 0.51 (0.40, 0.65) | 7.96E-08 |
| LPE(18:0) | HMDB0011129/LipidBlast454383 | Lipids and lipid-like molecules | Glycerophospholipids | 0.602 | 2.28E-12 | 0.777 | 7.74E-07 | 0.35 (0.26, 0.47) | 4.69E-12 |
| LPE(20:1) | HMDB0011482 | Lipids and lipid-like molecules | Glycerophospholipids | 0.626 | 1.18E-06 | 0.703 | 1.08E-05 | 0.47 (0.36, 0.61) | 2.01E-08 |
| LPG(18:1) | LipidBlast454445 | Lipids and lipid-like molecules | Glycerophospholipids | 0.577 | 2.28E-12 | 0.673 | 1.86E-07 | 0.37 (0.28, 0.49) | 1.01E-11 |
| LPI(18:1) | LipidBlast454506 | Lipids and lipid-like molecules | Glycerophospholipids | 0.667 | 6.77E-10 | 0.710 | 3.50E-08 | 0.32 (0.23, 0.44) | 9.36E-12 |
| LPI(20:3) | LipidBlast454517 | Lipids and lipid-like molecules | Glycerophospholipids | 0.781 | 1.35E-04 | 0.777 | 4.44E-06 | 0.45 (0.34, 0.59) | 5.46E-09 |
| LPI(20:4) | LipidBlast454518 | Lipids and lipid-like molecules | Glycerophospholipids | 0.776 | 5.95E-04 | 0.743 | 3.12E-06 | 0.42 (0.31, 0.57) | 4.17E-08 |
| Lutein | HMDB0003233 | Lipids and lipid-like molecules | Prenol lipids | 0.471 | 6.73E-11 | 0.675 | 2.85E-04 | 0.46 (0.35, 0.60) | 5.65E-09 |
| LysoPE(0:0/24:0) | HMDB0011497 | Lipids and lipid-like molecules | Glycerophospholipids | 0.576 | 4.99E-09 | 0.731 | 2.34E-04 | 0.49 (0.39, 0.63) | 1.12E-08 |
| LysoPE(20:1(11Z)/0:0) | HMDB0011512 | Lipids and lipid-like molecules | Glycerophospholipids | 0.547 | 1.14E-08 | 0.715 | 1.06E-04 | 0.46 (0.35, 0.60) | 7.92E-09 |
| LysoPI(18:0/0:0) | HMDB0240261 | Lipids and lipid-like molecules | Glycerophospholipids | 0.589 | 4.08E-07 | 0.734 | 1.37E-03 | 0.44 (0.33, 0.59) | 1.61E-08 |
| Maslinic acid | HMDB0002392 | Lipids and lipid-like molecules | Prenol lipids | 0.600 | 2.76E-07 | 0.673 | 6.27E-05 | 0.41 (0.31, 0.55) | 7.24E-10 |
| Methyl jasmonate | HMDB0036583 | Lipids and lipid-like molecules | Fatty acyls | 0.512 | 2.28E-12 | 0.452 | 4.67E-11 | 0.17 (0.10, 0.27) | 1.26E-13 |
| MG(0:0/15:0/0:0) | HMDB0011532 | Lipids and lipid-like molecules | Glycerolipids | 0.347 | 2.28E-12 | 0.554 | 3.53E-08 | 0.25 (0.17, 0.37) | 1.26E-12 |
| Momordicilin | HMDB0030896 | Lipids and lipid-like molecules | Prenol lipids | 0.393 | 2.28E-12 | 0.600 | 1.13E-06 | 0.35 (0.26, 0.47) | 4.84E-12 |
| Momordol | HMDB0029804 | Lipids and lipid-like molecules | Fatty acyls | 0.377 | 2.28E-12 | 0.455 | 4.67E-11 | 0.18 (0.11, 0.29) | 2.71E-13 |
| Myristoleic acid | HMDB0002000 | Lipids and lipid-like molecules | Fatty acyls | 0.437 | 2.28E-12 | 0.510 | 3.62E-04 | 0.40 (0.30, 0.52) | 2.93E-11 |
| Neoreticulatacin A | HMDB0034924 | Lipids and lipid-like molecules | Fatty acyls | 0.511 | 1.37E-09 | 0.632 | 2.18E-04 | 0.47 (0.36, 0.60) | 3.43E-09 |
| Oleamide | HMDB0002117 | Lipids and lipid-like molecules | Fatty acyls | 1.388 | 7.75E-05 | 1.414 | 3.98E-03 | 1.71 (1.37, 2.15) | 3.12E-06 |
| OxPI(18:0/18:1+3O) | Not available | Lipids and lipid-like molecules | Fatty acyls | 0.411 | 2.43E-12 | 0.648 | 3.32E-02 | 0.41 (0.30, 0.54) | 1.54E-09 |
| PC(14:0e/3:0) | Not available | Lipids and lipid-like molecules | Glycerophospholipids | 0.570 | 2.28E-12 | 0.734 | 5.51E-04 | 0.39 (0.29, 0.52) | 6.94E-10 |
| PC(14:0e/4:0) | Not available | Lipids and lipid-like molecules | Glycerophospholipids | 0.622 | 2.28E-12 | 0.767 | 1.17E-09 | 0.27 (0.19, 0.38) | 2.02E-13 |
| PC(16:2e/2:0) | Not available | Lipids and lipid-like molecules | Glycerophospholipids | 0.561 | 1.71E-08 | 0.756 | 2.76E-03 | 0.52 (0.41, 0.66) | 8.32E-08 |
| PC(18:5e/6:0) | Not available | Lipids and lipid-like molecules | Glycerophospholipids | 0.486 | 2.28E-12 | 0.776 | 4.28E-04 | 0.40 (0.31, 0.52) | 2.12E-11 |
| PC(2:0/20:2) | Not available | Lipids and lipid-like molecules | Glycerophospholipids | 0.527 | 6.02E-12 | 0.676 | 2.63E-07 | 0.35 (0.26, 0.48) | 1.05E-11 |
| PC(22:5/22:6) | HMDB0008682 | Lipids and lipid-like molecules | Glycerophospholipids | 0.426 | 2.28E-12 | 0.756 | 3.11E-02 | 0.44 (0.34, 0.58) | 3.23E-09 |
| PE(18:2e/2:0) | Not available | Lipids and lipid-like molecules | Glycerophospholipids | 0.797 | 3.00E-07 | 0.788 | 4.47E-07 | 0.45 (0.35, 0.59) | 5.66E-09 |
| PE(20:3e/3:0) | Not available | Lipids and lipid-like molecules | Glycerophospholipids | 0.390 | 2.28E-12 | 0.464 | 4.67E-11 | 0.09 (0.04, 0.18) | 7.08E-12 |
| Pentadecanoic acid | HMDB0000826 | Lipids and lipid-like molecules | Fatty acyls | 0.767 | 1.08E-06 | 0.793 | 6.53E-03 | 0.52 (0.41, 0.67) | 2.57E-07 |
| PGD2 ethanolamide | HMDB0013629 | Lipids and lipid-like molecules | Fatty acyls | 0.471 | 2.28E-12 | 0.765 | 1.41E-03 | 0.36 (0.27, 0.48) | 4.70E-12 |
| PI(16:0/18:1) | HMDB0009782 | Lipids and lipid-like molecules | Glycerophospholipids | 0.405 | 2.28E-12 | 0.748 | 2.41E-03 | 0.34 (0.26, 0.46) | 2.06E-12 |
| PI(16:0/18:3) | Not available | Lipids and lipid-like molecules | Glycerophospholipids | 0.369 | 2.28E-12 | 0.736 | 8.24E-06 | 0.30 (0.22, 0.42) | 2.57E-12 |
| PI(16:0/20:3) | HMDB0009787 | Lipids and lipid-like molecules | Glycerophospholipids | 0.382 | 2.28E-12 | 0.732 | 4.89E-06 | 0.31 (0.22, 0.42) | 4.02E-13 |
| PI(17:0/20:4) | Not available | Lipids and lipid-like molecules | Glycerophospholipids | 0.460 | 2.28E-12 | 0.794 | 2.56E-03 | 0.32 (0.23, 0.44) | 2.11E-12 |
| PI(18:0/18:1) | Not available | Lipids and lipid-like molecules | Glycerophospholipids | 0.551 | 2.28E-11 | 0.789 | 4.17E-02 | 0.46 (0.36, 0.60) | 2.97E-09 |
| PI(18:0/20:3) | Not available | Lipids and lipid-like molecules | Glycerophospholipids | 0.432 | 2.28E-12 | 0.668 | 3.52E-04 | 0.32 (0.24, 0.44) | 5.47E-13 |
| PI(18:0/20:4) | Not available | Lipids and lipid-like molecules | Glycerophospholipids | 0.523 | 2.28E-12 | 0.795 | 5.68E-03 | 0.38 (0.29, 0.51) | 6.55E-11 |
| PI(18:1/18:1) | HMDB0009824 | Lipids and lipid-like molecules | Glycerophospholipids | 0.460 | 2.28E-12 | 0.744 | 7.08E-03 | 0.36 (0.27, 0.48) | 2.11E-11 |
| PI(18:1/20:4) | HMDB0009832 | Lipids and lipid-like molecules | Glycerophospholipids | 0.337 | 2.28E-12 | 0.759 | 1.86E-05 | 0.24 (0.17, 0.35) | 6.60E-14 |
| PI(19:1/19:1) | Not available | Lipids and lipid-like molecules | Glycerophospholipids | 0.371 | 2.28E-12 | 0.571 | 3.15E-04 | 0.38 (0.28, 0.50) | 2.87E-11 |
| PI(2:0/14:1) | Not available | Lipids and lipid-like molecules | Glycerophospholipids | 0.680 | 5.78E-05 | 0.737 | 1.19E-02 | 0.57 (0.46, 0.72) | 1.27E-06 |
| PI(22:4(10Z,13Z,16Z,19Z)/16:0) | HMDB0009912 | Lipids and lipid-like molecules | Glycerophospholipids | 0.453 | 2.28E-12 | 0.769 | 3.00E-03 | 0.37 (0.27, 0.49) | 5.07E-11 |
| Polyoxyethylene (600) monoricinoleate | HMDB0032476 | Lipids and lipid-like molecules | Fatty acyls | 0.563 | 2.28E-12 | 0.747 | 4.53E-06 | 0.36 (0.26, 0.49) | 5.38E-11 |
| Ruscogenin | HMDB0257364 | Lipids and lipid-like molecules | Prenol lipids | 0.538 | 2.28E-12 | 0.740 | 1.51E-10 | 0.18 (0.12, 0.29) | 5.30E-14 |
| Setariol | HMDB0041583 | Lipids and lipid-like molecules | Steroids and steroid derivatives | 0.357 | 2.28E-12 | 0.509 | 4.67E-11 | 0.23 (0.16, 0.34) | 2.90E-14 |
| SHexCer(d28:1) | Not available | Lipids and lipid-like molecules | Fatty acyls | 0.684 | 8.22E-06 | 0.750 | 1.07E-05 | 0.50 (0.38, 0.65) | 1.48E-07 |
| SQDG(12:0/13:0) | Not available | Lipids and lipid-like molecules | Glycerolipids | 0.633 | 1.17E-05 | 0.646 | 1.19E-05 | 0.51 (0.40, 0.65) | 5.96E-08 |
| Stearidonic acid | HMDB0006547 | Lipids and lipid-like molecules | Fatty acyls | 0.591 | 4.24E-11 | 0.694 | 3.96E-06 | 0.42 (0.32, 0.55) | 3.09E-10 |
| Stearoylcarnitine | HMDB0000848 | Lipids and lipid-like molecules | Fatty acyls | 0.657 | 2.28E-12 | 0.785 | 3.06E-06 | 0.36 (0.27, 0.49) | 1.88E-11 |
| TAG(22:3/22:7/22:7) | Not available | Lipids and lipid-like molecules | Glycerolipids | 0.444 | 2.28E-12 | 0.634 | 5.68E-08 | 0.27 (0.19, 0.39) | 1.20E-12 |
| TAG(22:5/22:7/22:7) | Not available | Lipids and lipid-like molecules | Glycerolipids | 0.673 | 1.37E-04 | 0.782 | 1.02E-03 | 0.53 (0.41, 0.69) | 1.62E-06 |
| TAG(22:6/22:7/22:7) | Not available | Lipids and lipid-like molecules | Glycerolipids | 0.663 | 2.28E-12 | 0.792 | 1.97E-04 | 0.39 (0.29, 0.52) | 2.60E-10 |
| TAG(22:7/22:7/22:7) | Not available | Lipids and lipid-like molecules | Glycerolipids | 0.670 | 6.85E-04 | 0.743 | 5.49E-03 | 0.50 (0.37, 0.67) | 3.35E-06 |
| Tridecanoic acid | HMDB0000910 | Lipids and lipid-like molecules | Fatty acyls | 0.777 | 8.12E-06 | 0.734 | 9.16E-03 | 0.52 (0.40, 0.68) | 1.47E-06 |
| Ubiquinone-1 | HMDB0002012 | Lipids and lipid-like molecules | Prenol lipids | 0.546 | 1.38E-09 | 0.736 | 4.32E-05 | 0.38 (0.28, 0.51) | 1.27E-10 |
| Undecylenic acid | HMDB0033724 | Lipids and lipid-like molecules | Fatty acyls | 0.560 | 2.28E-12 | 0.600 | 1.70E-08 | 0.31 (0.23, 0.42) | 3.59E-13 |
| (10)-Gingerol | HMDB0033616 | Benzenoids | Phenols | 0.579 | 2.28E-12 | 0.537 | 2.91E-10 | 0.25 (0.17, 0.37) | 2.00E-12 |
| [12]-Gingerol | HMDB0036356 | Benzenoids | Phenols | 0.564 | 2.28E-12 | 0.609 | 4.67E-11 | 0.24 (0.17, 0.35) | 4.60E-14 |
| [2,2-Bis(2-methylpropoxy)ethyl]benzene | HMDB0037712 | Benzenoids | Benzene and substituted derivatives | 0.301 | 2.28E-12 | 0.460 | 4.67E-11 | 0.19 (0.12, 0.29) | 9.30E-14 |
| [7]-Paradol | HMDB0040806 | Benzenoids | Phenols | 0.748 | 1.97E-05 | 0.721 | 2.26E-07 | 0.47 (0.36, 0.60) | 4.57E-09 |
| [8]-Paradol | HMDB0040640 | Benzenoids | Phenols | 1.498 | 1.78E-05 | 1.414 | 2.90E-02 | 1.65 (1.31, 2.07) | 1.66E-05 |
| 2-Isopropylphenyl methylcarbamate | HMDB0031797 | Benzenoids | Benzene and substituted derivatives | 0.736 | 3.55E-12 | 0.761 | 9.27E-09 | 0.32 (0.23, 0.45) | 2.08E-11 |
| 2-Methyl-1-phenyl-2-propanyl butyrate | HMDB0040226 | Benzenoids | Benzene and substituted derivatives | 0.400 | 2.28E-12 | 0.509 | 4.67E-11 | 0.23 (0.15, 0.33) | 4.20E-14 |
| 3,4-Dihydroxybenzoic acid | HMDB0001856 | Benzenoids | Benzene and substituted derivatives | 0.394 | 2.28E-12 | 0.436 | 4.67E-11 | 0.24 (0.16, 0.35) | 1.60E-13 |
| Butyl 2-aminobenzoate | HMDB0036906 | Benzenoids | Benzene and substituted derivatives | 0.736 | 4.15E-08 | 0.786 | 9.70E-06 | 0.42 (0.32, 0.55) | 6.32E-10 |
| Ethiofencarb | HMDB0031782 | Benzenoids | Benzene and substituted derivatives | 0.744 | 1.14E-09 | 0.743 | 5.58E-11 | 0.33 (0.24, 0.45) | 1.30E-11 |
| Ethylbenzene | HMDB0059905 | Benzenoids | Benzene and substituted derivatives | 0.770 | 9.10E-04 | 0.732 | 1.30E-03 | 0.57 (0.45, 0.72) | 1.97E-06 |
| Eugenol | HMDB0005809 | Benzenoids | Phenols | 0.767 | 6.00E-05 | 0.786 | 3.20E-04 | 0.51 (0.40, 0.65) | 5.69E-08 |
| Ginkgoic acid | HMDB0033897 | Benzenoids | Benzene and substituted derivatives | 0.487 | 2.28E-12 | 0.492 | 2.01E-07 | 0.28 (0.20, 0.40) | 3.03E-13 |
| Methyl 3-(2,3-dihydroxy-3-methylbutyl)-4-hydroxybenzoate | HMDB0032796 | Benzenoids | Benzene and substituted derivatives | 0.565 | 7.92E-09 | 0.595 | 1.48E-07 | 0.41 (0.32, 0.54) | 4.26E-11 |
| o-Vinylanisole | HMDB0036441 | Benzenoids | Phenol ethers | 0.732 | 1.17E-03 | 0.781 | 1.91E-03 | 0.63 (0.52, 0.78) | 1.35E-05 |
| Toluene | HMDB0034168 | Benzenoids | Benzene and substituted derivatives | 0.746 | 2.74E-05 | 0.759 | 4.85E-03 | 0.59 (0.47, 0.72) | 9.11E-07 |
| 2,2,6,7-Tetramethylbicyclo[4.3.0]nona-1(9),4-dien-8-one | MDB0036685 | Organic oxygen compounds | Organooxygen compounds | 0.495 | 1.69E-09 | 0.742 | 7.14E-04 | 0.51 (0.41, 0.64) | 6.87E-09 |
| 2-trans-6-cis-Dodecadienal | HMDB0032531 | Organic oxygen compounds | Organooxygen compounds | 0.356 | 2.28E-12 | 0.438 | 4.67E-11 | 0.21 (0.14, 0.31) | 3.30E-14 |
| 6-(2-Carboxyethyl)-7-hydroxy-2,2-dimethyl-4-chromanone glucoside | HMDB0034032 | Organic oxygen compounds | Organooxygen compounds | 0.531 | 2.28E-12 | 0.591 | 2.74E-08 | 0.28 (0.20, 0.40) | 1.56E-12 |
| Cer/AP(t14:2/13:1) | Not available | Organic oxygen compounds | Organooxygen compounds | 0.427 | 2.28E-12 | 0.531 | 4.67E-11 | 0.20 (0.13, 0.30) | 1.90E-14 |
| Cer/AP(t14:2/19:1) | Not available | Organic oxygen compounds | Organooxygen compounds | 0.564 | 1.24E-10 | 0.593 | 5.94E-10 | 0.30 (0.21, 0.42) | 2.70E-12 |
| Cer/AP(t15:1/16:2) | Not available | Organic oxygen compounds | Organooxygen compounds | 0.549 | 3.02E-12 | 0.568 | 1.82E-09 | 0.31 (0.23, 0.43) | 3.33E-12 |
| Cer/AP(t15:2/16:2) | Not available | Organic oxygen compounds | Organooxygen compounds | 0.642 | 7.05E-12 | 0.641 | 5.81E-11 | 0.28 (0.19, 0.40) | 1.72E-12 |
| Cer/AP(t15:2/18:2) | Not available | Organic oxygen compounds | Organooxygen compounds | 0.589 | 6.54E-09 | 0.556 | 1.35E-07 | 0.37 (0.27, 0.50) | 2.54E-10 |
| Cer/AP(t15:2/20:2) | Not available | Organic oxygen compounds | Organooxygen compounds | 0.645 | 3.34E-10 | 0.647 | 6.31E-09 | 0.36 (0.27, 0.49) | 1.39E-11 |
| Cer/AP(t17:2/12:1) | Not available | Organic oxygen compounds | Organooxygen compounds | 0.694 | 3.98E-09 | 0.795 | 3.37E-06 | 0.32 (0.22, 0.46) | 1.81E-09 |
| Cer/AS(d14:2/13:1) | Not available | Organic oxygen compounds | Organooxygen compounds | 0.492 | 2.28E-12 | 0.464 | 4.67E-11 | 0.20 (0.13, 0.30) | 4.70E-14 |
| Cer/AS(d14:3/13:1) | Not available | Organic oxygen compounds | Organooxygen compounds | 0.545 | 2.28E-12 | 0.586 | 4.67E-11 | 0.26 (0.18, 0.38) | 3.67E-13 |
| Cer/AS(d15:3/16:2) | Not available | Organic oxygen compounds | Organooxygen compounds | 0.714 | 5.08E-04 | 0.763 | 7.03E-04 | 0.57 (0.45, 0.72) | 2.12E-06 |
| D-Maltose | HMDB0000163 | Organic oxygen compounds | Organooxygen compounds | 7.373 | 2.28E-12 | 1.631 | 1.53E-04 | 3.28 (2.34, 4.58) | 3.91E-12 |
| D-Xylulose | HMDB0001644 | Organic oxygen compounds | Carbohydrates and carbohydrate conjugates | 1.256 | 2.50E-10 | 1.730 | 3.58E-10 | 2.54 (1.88, 3.43) | 1.53E-09 |
| Methyl lucidenate F | HMDB0036437 | Organic oxygen compounds | Prenol lipids | 0.567 | 3.91E-11 | 0.763 | 1.89E-03 | 0.45 (0.35, 0.58) | 3.68E-10 |
| Raffinose | HMDB0003213 | Organic oxygen compounds | Organooxygen compounds | 7.783 | 2.28E-12 | 1.352 | 2.28E-03 | 2.65 (1.94, 3.62) | 8.52E-10 |
| Sucrose | HMDB0000258 | Organic oxygen compounds | Organooxygen compounds | 8.802 | 2.28E-12 | 1.268 | 9.16E-03 | 3.00 (2.16, 4.18) | 6.91E-11 |
| 2-Ketobutyric acid | HMDB0000005 | Organic acids and derivatives | Keto acids and derivatives | 1.240 | 1.19E-06 | 1.643 | 6.17E-10 | 2.12 (1.64, 2.73) | 7.07E-09 |
| 3-Hydroxydecanoic acid | HMDB0002203 | Organic acids and derivatives | Hydroxy acids and derivatives | 0.521 | 2.28E-12 | 0.644 | 6.47E-08 | 0.32 (0.24, 0.43) | 2.82E-13 |
| 4-Methyl-2-oxovaleric acid | HMDB0000695 | Organic acids and derivatives | Keto acids and derivatives | 0.787 | 7.30E-08 | 0.753 | 4.81E-08 | 0.35 (0.26, 0.48) | 3.47E-11 |
| Alpha-N-Phenylacetyl-L-glutamine | HMDB0006344 | Organic acids and derivatives | Carboxylic acids and derivatives | 3.388 | 2.09E-06 | 2.867 | 2.75E-08 | 2.45 (1.87, 3.21) | 8.63E-11 |
| Glutaric acid | HMDB0000661 | Organic acids and derivatives | Carboxylic acids and derivatives | 1.239 | 2.95E-10 | 1.706 | 1.46E-07 | 2.11 (1.62, 2.75) | 3.78E-08 |
| Isomultiflorenyl acetate | HMDB0038062 | Organic acids and derivatives | Carboxylic acids and derivatives | 0.354 | 2.28E-12 | 0.498 | 4.67E-11 | 0.11 (0.06, 0.20) | 4.24E-12 |
| LDGTS(16:4) | LipidBlast2022_444989 | Organic acids and derivatives | Carboxylic acids and derivatives | 0.577 | 4.97E-09 | 0.783 | 4.33E-03 | 0.47 (0.37, 0.61) | 2.40E-09 |
| N-Isovaleroylglycine | HMDB0000678 | Organic acids and derivatives | Carboxylic acids and derivatives | 1.524 | 2.60E-05 | 1.492 | 1.51E-02 | 1.72 (1.37, 2.17) | 3.18E-06 |
| N-Ornithyl-L-taurine | HMDB0033519 | Organic acids and derivatives | Carboxylic acids and derivatives | 0.395 | 2.28E-12 | 0.629 | 2.29E-06 | 0.36 (0.27, 0.48) | 1.87E-12 |
| N-Salicyloylaspartic acid | HMDB0039506 | Organic acids and derivatives | Carboxylic acids and derivatives | 0.497 | 1.20E-08 | 0.397 | 4.67E-11 | 0.26 (0.17, 0.38) | 3.87E-11 |
| O-Ureidohomoserine | HMDB0012271 | Organic acids and derivatives | Carboxylic acids and derivatives | 0.684 | 6.96E-08 | 0.659 | 1.09E-07 | 0.39 (0.29, 0.51) | 5.24E-11 |
| Oxoadipic acid | HMDB0000225 | Organic acids and derivatives | Keto acids and derivatives | 1.207 | 4.25E-02 | 1.754 | 5.01E-08 | 1.92 (1.51, 2.44) | 1.34E-07 |
| 2-Butylfuran | HMDB0040272 | Organoheterocyclic compounds | Heteroaromatic compounds | 0.775 | 1.62E-10 | 0.794 | 2.70E-09 | 0.34 (0.25, 0.47) | 2.26E-11 |
| 2-Hydroxyxanthone | HMDB0032997 | Organoheterocyclic compounds | Benzopyrans | 0.583 | 2.28E-12 | 0.564 | 4.67E-11 | 0.31 (0.22, 0.42) | 1.17E-13 |
| 4-Hydroxy-2H-pyran-3-carboxaldehyde | HMDB0031256 | Organoheterocyclic compounds | Organoheterocyclic compounds | 1.312 | 3.15E-05 | 1.973 | 1.18E-09 | 2.08 (1.63, 2.66) | 3.62E-09 |
| 5-Hydroxy-L-Tryptophan | HMDB0015571 | Organoheterocyclic compounds | Indoles and derivatives | 0.679 | 6.36E-05 | 0.726 | 6.73E-08 | 0.50 (0.40, 0.63) | 3.55E-09 |
| 5-Pentyl-3h-furan-2-one | HMDB0032463 | Organoheterocyclic compounds | Oxolanes | 0.681 | 5.17E-10 | 0.687 | 6.80E-09 | 0.42 (0.32, 0.54) | 1.33E-11 |
| Caffeine | HMDB0001847 | Organoheterocyclic compounds | Imidazopyrimidines | 0.279 | 1.72E-04 | 0.327 | 1.64E-04 | 0.56 (0.44, 0.71) | 8.90E-07 |
| Furanone A | HMDB0094691 | Organoheterocyclic compounds | Dihydrofurans | 1.389 | 5.62E-09 | 1.465 | 1.17E-07 | 2.75 (2.04, 3.71) | 3.02E-11 |
| Isosalsolidine | HMDB0041526 | Organoheterocyclic compounds | Isoquinolines and derivatives | 0.635 | 3.94E-12 | 0.736 | 2.14E-08 | 0.27 (0.18, 0.40) | 4.96E-11 |
| Laccarin | HMDB0041440 | Organoheterocyclic compounds | Pyrrolopyridines | 0.577 | 2.28E-12 | 0.754 | 3.95E-06 | 0.34 (0.25, 0.47) | 1.20E-11 |
| Phaeophorbide b | HMDB0031149 | Organoheterocyclic compounds | Tetrapyrroles and derivatives | 0.786 | 3.58E-04 | 0.659 | 2.78E-04 | 0.52 (0.41, 0.66) | 1.88E-07 |
| xi-5-Dodecanolide | HMDB0037742 | Organoheterocyclic compounds | Lactones | 0.389 | 2.28E-12 | 0.547 | 2.90E-09 | 0.25 (0.18, 0.36) | 3.40E-14 |
| Biochanin A | HMDB0002338 | Phenylpropanoids and polyketides | Isoflavonoids | 0.777 | 5.47E-04 | 0.785 | 1.44E-02 | 0.56 (0.44, 0.72) | 4.17E-06 |
| Hypoletin 8-gentiobioside | HMDB0038824 | Phenylpropanoids and polyketides | Flavonoids | 0.483 | 2.28E-12 | 0.747 | 3.63E-09 | 0.20 (0.13, 0.31) | 6.00E-14 |
| Kuwanon V | HMDB0030115 | Phenylpropanoids and polyketides | Diarylheptanoids | 0.395 | 2.28E-12 | 0.550 | 4.67E-11 | 0.22 (0.15, 0.33) | 3.72E-13 |
| Mulberrofuran E | HMDB0041423 | Phenylpropanoids and polyketides | 2-arylbenzofuran flavonoids | 0.208 | 2.28E-12 | 0.530 | 7.64E-11 | 0.22 (0.15, 0.33) | 7.20E-14 |
| Trans-2, 3, 4-Trimethoxycinnamate | HMDB0011721 | Phenylpropanoids and polyketides | Cinnamic acids and derivatives | 0.497 | 1.79E-08 | 0.646 | 1.03E-02 | 0.48 (0.36, 0.63) | 1.89E-07 |
| 5-Methylcytidine | HMDB0000982 | Nucleosides, nucleotides, and analogues | Pyrimidine nucleosides | 0.510 | 9.08E-08 | 0.626 | 4.37E-04 | 0.44 (0.33, 0.59) | 3.33E-08 |
| Adenosine 3',5'-diphosphate | HMDB0000061 | Nucleosides, nucleotides, and analogues | Purine nucleotides | 0.381 | 2.28E-12 | 0.508 | 3.57E-10 | 0.21 (0.14, 0.32) | 2.89E-13 |
| Adenosine monophosphate (AMP) | HMDB0000045 | Nucleosides, nucleotides, and analogues | Purine nucleotides | 0.535 | 1.59E-08 | 0.641 | 1.75E-07 | 0.33 (0.23, 0.48) | 2.31E-09 |
| ADP | HMDB0001341 | Nucleosides, nucleotides, and analogues | Nucleosides, nucleotides, and analogues | 0.428 | 2.28E-12 | 0.494 | 6.18E-10 | 0.17 (0.10, 0.29) | 1.93E-11 |
| 2-(5-Methyl-2-furanyl)pyrrolidine | HMDB0040044 | Organic nitrogen compounds | Amines | 0.755 | 2.52E-03 | 0.755 | 1.18E-04 | 0.55 (0.43, 0.70) | 9.68E-07 |
| Beta-Guanidinopropionic acid | HMDB0013222 | Organic nitrogen compounds | Organonitrogen compounds | 1.363 | 9.40E-04 | 1.433 | 3.89E-04 | 2.17 (1.63, 2.88) | 8.60E-08 |
| APC | HMDB0060661 | Alkaloids and derivatives | Camptothecins | 0.742 | 2.42E-08 | 0.789 | 1.82E-05 | 0.41 (0.31, 0.54) | 4.21E-10 |
| **CRA vs. NC** |  |  |  |  |  |  |  |  |  |
| (9S,10E,12Z,15Z)-9-Hydroxy-10,12,15-octadecatrienoic acid | HMDB0031934 | Lipids and lipid-like molecules | Lineolic acids and derivatives | 0.550 | 2.18E-06 | 0.576 | 2.88E-05 | 0.42 (0.28,0.63) | 6.35E-05 |
| ACar(10:2) | LipidBlast000014 | Lipids and lipid-like molecules | Fatty acyls | 0.774 | 4.34E-03 | 0.605 | 1.89E-04 | 0.60 (0.42, 0.85) | 4.51E-03 |
| ACar(18:3) | LipidBlast000044 | Lipids and lipid-like molecules | Fatty acyls | 0.717 | 4.24E-03 | 0.638 | 8.93E-07 | 0.40 (0.26,0.62) | 5.87E-05 |
| HexCer/NS(d16:1/16:0) | Not available | Lipids and lipid-like molecules | Fatty acyls | 0.337 | 3.61E-11 | 0.687 | 1.68E-04 | 0.20 (0.12,0.36) | 9.40E-07 |
| HexCer/NS(d18:2/16:0) | Not available | Lipids and lipid-like molecules | Fatty acyls | 0.385 | 3.61E-11 | 0.694 | 9.59E-04 | 0.27 (0.15,0.47) | 4.55E-06 |
| Palmitic acid | HMDB0000220 | Lipids and lipid-like molecules | Fatty acyls | 0.353 | 4.45E-11 | 0.130 | 1.18E-10 | 0.09 (0.04,0.20) | 3.22E-06 |
| PC(22:5/22:6) | HMDB0008682 | Lipids and lipid-like molecules | Glycerophospholipids | 0.416 | 1.28E-08 | 0.776 | 2.98E-02 | 0.44 (0.29,0.66) | 7.70E-05 |
| PC(22:6(4Z,7Z,10Z,13Z,16Z,19Z)/20:5(5Z,8Z,11Z,14Z,17Z)) | HMDB0008741 | Lipids and lipid-like molecules | Glycerophospholipids | 0.402 | 5.50E-11 | 0.714 | 9.04E-05 | 0.29 (0.17,0.49) | 4.10E-06 |
| PI(16:0/18:2) | HMDB0009784 | Lipids and lipid-like molecules | Glycerophospholipids | 0.303 | 2.60E-10 | 0.795 | 3.30E-02 | 0.38 (0.24,0.61) | 6.29E-05 |
| PI(16:0/18:3) | Not available | Lipids and lipid-like molecules | Glycerophospholipids | 0.319 | 5.49E-11 | 0.657 | 2.74E-04 | 0.32 (0.19,0.55) | 3.97E-05 |
| PI(16:0/20:3) | HMDB0009787 | Lipids and lipid-like molecules | Glycerophospholipids | 0.346 | 1.40E-10 | 0.670 | 1.92E-06 | 0.33 (0.20,0.56) | 2.52E-05 |
| PI(18:1/20:4) | HMDB0009832 | Lipids and lipid-like molecules | Glycerophospholipids | 0.279 | 3.61E-11 | 0.696 | 2.52E-05 | 0.17 (0.08,0.33) | 5.20E-07 |
| Prostaglandin B1 | HMDB0002982 | Lipids and lipid-like molecules | Fatty acyls | 0.688 | 1.75E-04 | 0.677 | 3.12E-03 | 0.47 (0.31,0.72) | 5.93E-04 |
| Setariol | HMDB0041583 | Lipids and lipid-like molecules | Steroids and steroid derivatives | 0.467 | 1.39E-08 | 0.447 | 6.29E-10 | 0.12 (0.06,0.25) | 8.90E-07 |
| SHexCer(d29:1) | Not available | Lipids and lipid-like molecules | Fatty acyls | 0.224 | 3.61E-11 | 0.504 | 5.14E-04 | 0.49 (0.35,0.68) | 3.35E-05 |
| (R)-Pelletierine | HMDB0030324 | Organoheterocyclic compounds | Piperidines | 0.786 | 1.57E-02 | 0.793 | 1.38E-02 | 0.65 (0.45,0.94) | 2.56E-02 |
| 2-Hydroxyxanthone | HMDB0032997 | Organoheterocyclic compounds | Benzopyrans | 0.763 | 2.01E-02 | 0.734 | 2.50E-04 | 0.44 (0.29,0.68) | 2.73E-04 |
| 5-Hydroxy-L-Tryptophan | HMDB0015571 | Organoheterocyclic compounds | Indoles and derivatives | 0.594 | 9.63E-05 | 0.715 | 1.25E-02 | 0.48 (0.33,0.70) | 1.82E-04 |
| 5-Pentyl-3h-furan-2-one | HMDB0032463 | Organoheterocyclic compounds | Oxolanes | 0.686 | 4.69E-06 | 0.725 | 4.67E-05 | 0.44 (0.30,0.66) | 1.11E-04 |
| I-Urobilin | HMDB0004160 | Organoheterocyclic compounds | Tetrapyrroles and derivatives | 3.329 | 4.19E-03 | 1.871 | 1.64E-02 | 1.60 (1.15,2.23) | 5.50E-03 |
| Oleoyl glycine | HMDB0013631 | Organic acids and derivatives | Carboxylic acids and derivatives | 0.533 | 1.15E-04 | 0.104 | 1.18E-10 | 0.12 (0.06,0.25) | 1.59E-06 |
| Palmitoylethanolamide | HMDB0002100 | Organic acids and derivatives | Carboximidic acids and derivatives | 0.575 | 5.29E-03 | 0.378 | 1.18E-10 | 0.32 (0.20,0.51) | 3.52E-06 |
| Methyl 3-(2,3-dihydroxy-3-methylbutyl)-4-hydroxybenzoate | HMDB0032796 | Benzenoids | Benzene and substituted derivatives | 0.603 | 6.06E-04 | 0.726 | 2.15E-02 | 0.56 (0.39,0.80) | 1.86E-03 |
| Cyclotetradecane | HMDB0033567 | Hydrocarbons | Saturated hydrocarbons | 2.854 | 2.66E-02 | 1.379 | 2.45E-02 | 2.80 (1.75,4.47) | 1.11E-04 |
| Phytosphingosine | HMDB0004610 | Organic nitrogen compounds | Organonitrogen compounds | 0.363 | 2.60E-10 | 0.028 | 1.18E-10 | 0.07 (0.02,0.19) | 1.82E-04 |
| 2,2,6,7-Tetramethylbicyclo[4.3.0]nona-1(9),4-dien-8-one |  | Organic oxygen compounds | Organooxygen compounds | 0.601 | 8.47E-05 | 0.780 | 4.01E-02 | 0.51 (0.35,0.76) | 7.55E-04 |
| **CRC vs. CRA** |  |  |  |  |  |  |  |  |  |
| (10betaH,11xi)-11-Hydroxy-13-nor-6-eremophilen-8-one | HMDB0037605 | Lipids and lipid-like molecules | Prenol lipids | 0.434 | 2.36E-11 | 0.628 | 3.63E-08 | 0.29 (0.18, 0.47) | 5.10E-07 |
| (10E,12Z)-9-HODE | HMDB0062652 | Lipids and lipid-like molecules | Fatty acyls | 0.667 | 2.81E-04 | 0.663 | 6.40E-06 | 0.43 (0.29, 0.66) | 7.60E-05 |
| (3beta,5alpha,6beta,7alpha,22E,24R)-Ergosta-8,22-diene-3,5,6,7-tetrol | HMDB0032107 | Lipids and lipid-like molecules | Steroids and steroid derivatives | 0.406 | 2.36E-11 | 0.570 | 1.40E-08 | 0.38 (0.26, 0.55) | 5.00E-07 |
| (9S,10S)-9,10-dihydroxyoctadecanoate | HMDB0059633 | Lipids and lipid-like molecules | Fatty acyls | 0.498 | 1.16E-06 | 0.682 | 2.54E-03 | 0.53 (0.38, 0.74) | 2.40E-04 |
| (R)-3-Hydroxy-tetradecanoic acid | HMDB0010731 | Lipids and lipid-like molecules | Fatty acyls | 0.766 | 5.96E-03 | 0.575 | 8.78E-10 | 0.52 (0.36, 0.75) | 5.05E-04 |
| 12,13-DHOME | HMDB0004705 | Lipids and lipid-like molecules | Fatty acyls | 0.495 | 8.85E-06 | 0.626 | 3.52E-03 | 0.61 (0.44, 0.84) | 2.50E-03 |
| 12,13-EpOME | HMDB0004702 | Lipids and lipid-like molecules | Fatty acyls | 0.519 | 6.98E-08 | 0.699 | 1.23E-02 | 0.50 (0.35, 0.73) | 2.58E-04 |
| 24-Epibrassinolide | HMDB0041130 | Lipids and lipid-like molecules | Steroids and steroid derivatives | 0.472 | 2.36E-11 | 0.587 | 6.24E-11 | 0.23 (0.14, 0.40) | 1.30E-07 |
| 3-Oxocholic acid | HMDB0000502 | Lipids and lipid-like molecules | Steroids and steroid derivatives | 0.498 | 4.63E-09 | 0.593 | 2.66E-08 | 0.31 (0.20, 0.51) | 2.28E-06 |
| 5-Oxooctadecanoic acid | HMDB0034074 | Lipids and lipid-like molecules | Fatty acyls | 0.523 | 1.52E-06 | 0.624 | 2.13E-03 | 0.58 (0.42, 0.79) | 6.04E-04 |
| 7-Ketocholesterol | HMDB0000501 | Lipids and lipid-like molecules | Steroids and steroid derivatives | 0.417 | 2.70E-11 | 0.589 | 2.84E-09 | 0.25 (0.15, 0.44) | 1.15E-06 |
| 9-Decenoic acid | HMDB0031003 | Lipids and lipid-like molecules | Fatty acyls | 0.747 | 3.54E-03 | 0.636 | 2.56E-06 | 0.53 (0.36, 0.79) | 1.46E-03 |
| 9-HODE | HMDB0010223 | Lipids and lipid-like molecules | Fatty acyls | 0.495 | 1.42E-06 | 0.609 | 5.07E-04 | 0.54 (0.38, 0.77) | 5.04E-04 |
| ACar(10:0) | LipidBlast000012 | Lipids and lipid-like molecules | Fatty acyls | 0.569 | 1.65E-06 | 0.577 | 7.09E-06 | 0.46 (0.31, 0.70) | 2.39E-04 |
| ACar(10:1) | LipidBlast000013 | Lipids and lipid-like molecules | Fatty acyls | 0.532 | 2.75E-08 | 0.673 | 1.53E-03 | 0.45 (0.30, 0.68) | 1.62E-04 |
| ACar(12:0) | LipidBlast000017 | Lipids and lipid-like molecules | Fatty acyls | 0.680 | 2.18E-04 | 0.587 | 4.43E-06 | 0.53 (0.36, 0.78) | 1.13E-03 |
| ACar(12:1) | LipidBlast000018 | Lipids and lipid-like molecules | Fatty acyls | 0.647 | 3.73E-06 | 0.675 | 1.18E-04 | 0.52 (0.36, 0.76) | 6.00E-04 |
| ACar(12:2) | LipidBlast000019 | Lipids and lipid-like molecules | Fatty acyls | 0.560 | 7.54E-07 | 0.703 | 2.54E-03 | 0.62 (0.45, 0.87) | 4.83E-03 |
| ACar(15:1) | LipidBlast000028 | Lipids and lipid-like molecules | Fatty acyls | 0.753 | 1.21E-04 | 0.774 | 4.82E-03 | 0.53 (0.36, 0.77) | 8.72E-04 |
| ACar(16:4) | LipidBlast000035 | Lipids and lipid-like molecules | Fatty acyls | 0.764 | 9.15E-03 | 0.735 | 7.89E-04 | 0.52 (0.35, 0.76) | 7.78E-04 |
| ACar(18:4) | LipidBlast000045 | Lipids and lipid-like molecules | Fatty acyls | 0.705 | 7.42E-06 | 0.729 | 7.87E-06 | 0.52 (0.35, 0.77) | 1.15E-03 |
| ACar(6:0) | LipidBlast000003 | Lipids and lipid-like molecules | Fatty acyls | 0.656 | 8.14E-06 | 0.710 | 2.41E-04 | 0.50 (0.33, 0.74) | 6.73E-04 |
| ACar(8:0) | LipidBlast000007 | Lipids and lipid-like molecules | Fatty acyls | 0.583 | 1.76E-05 | 0.609 | 2.49E-05 | 0.49 (0.32, 0.74) | 6.76E-04 |
| Cholestane-3,7,12,24,25-pentol | HMDB0002208 | Lipids and lipid-like molecules | Steroids and steroid derivatives | 0.392 | 2.88E-11 | 0.619 | 4.36E-05 | 0.44 (0.31, 0.63) | 1.05E-05 |
| Cholic acid | HMDB0000619 | Lipids and lipid-like molecules | Steroids and steroid derivatives | 0.526 | 3.37E-11 | 0.642 | 1.74E-06 | 0.36 (0.24, 0.55) | 1.65E-06 |
| DG(18:3(9Z,12Z,15Z)/15:0/0:0) | HMDB0007300 | Lipids and lipid-like molecules | Glycerolipids | 0.536 | 7.54E-07 | 0.706 | 1.10E-03 | 0.54 (0.38, 0.77) | 5.79E-04 |
| DG(18:4(6Z,9Z,12Z,15Z)/15:0/0:0) | HMDB0007329 | Lipids and lipid-like molecules | Glycerolipids | 0.450 | 6.25E-08 | 0.776 | 4.74E-02 | 0.49 (0.33, 0.72) | 2.89E-04 |
| DGTS(2:0/16:3) | Not available | Lipids and lipid-like molecules | Glycerolipids | 0.687 | 1.65E-04 | 0.756 | 7.51E-04 | 0.48 (0.34, 0.69) | 7.64E-05 |
| DGTS(2:0/20:1) | Not available | Lipids and lipid-like molecules | Glycerolipids | 0.649 | 3.80E-03 | 0.555 | 6.73E-04 | 0.50 (0.34, 0.71) | 1.65E-04 |
| DGTS(2:0/20:3) | Not available | Lipids and lipid-like molecules | Glycerolipids | 0.616 | 7.57E-03 | 0.728 | 1.76E-02 | 0.49 (0.33, 0.72) | 2.59E-04 |
| Dolichosterone | HMDB0034336 | Lipids and lipid-like molecules | Steroids and steroid derivatives | 0.470 | 2.42E-11 | 0.571 | 4.40E-10 | 0.26 (0.16, 0.44) | 3.10E-07 |
| Esculentic acid (Diplazium) | HMDB0035782 | Lipids and lipid-like molecules | Prenol lipids | 0.594 | 3.49E-09 | 0.752 | 2.15E-03 | 0.43 (0.28, 0.64) | 3.70E-05 |
| FA(21:3) | LipidBlast417815 | Lipids and lipid-like molecules | Fatty acyls | 0.452 | 1.27E-08 | 0.554 | 9.69E-09 | 0.37 (0.25, 0.56) | 3.10E-06 |
| FAHFA(2:0/22:2) | Not available | Lipids and lipid-like molecules | Fatty acyls | 0.601 | 4.25E-10 | 0.707 | 1.36E-06 | 0.30 (0.18, 0.49) | 1.72E-06 |
| Furanofukinin | HMDB0036640 | Lipids and lipid-like molecules | Prenol lipids | 0.377 | 4.08E-11 | 0.634 | 6.40E-06 | 0.35 (0.23, 0.53) | 1.05E-06 |
| Ganoderiol H | HMDB0037783 | Lipids and lipid-like molecules | Prenol lipids | 0.434 | 2.57E-11 | 0.681 | 1.76E-06 | 0.25 (0.14, 0.44) | 1.17E-06 |
| Glycerol triundecanoate | HMDB0031089 | Lipids and lipid-like molecules | Glycerolipids | 0.539 | 6.23E-07 | 0.730 | 7.64E-03 | 0.57 (0.42, 0.79) | 5.93E-04 |
| Goshuyic acid | HMDB0000560 | Lipids and lipid-like molecules | Fatty acyls | 0.560 | 5.31E-04 | 0.569 | 5.60E-07 | 0.50 (0.35, 0.70) | 5.77E-05 |
| HexCer/NS(d14:1/12:1) | Not available | Lipids and lipid-like molecules | Fatty acyls | 0.670 | 1.96E-05 | 0.795 | 1.92E-02 | 0.50 (0.35, 0.71) | 1.13E-04 |
| HexCer/NS(d14:2/16:2) | Not available | Lipids and lipid-like molecules | Fatty acyls | 0.630 | 1.20E-05 | 0.716 | 1.90E-02 | 0.57 (0.39, 0.82) | 2.71E-03 |
| HexCer/NS(d14:3/14:1) | Not available | Lipids and lipid-like molecules | Fatty acyls | 0.555 | 3.87E-07 | 0.717 | 6.81E-03 | 0.40(0.26, 0.61) | 2.74E-05 |
| Isothankunic acid | HMDB0040772 | Lipids and lipid-like molecules | Prenol lipids | 0.557 | 4.08E-11 | 0.712 | 2.26E-05 | 0.41 (0.27, 0.63) | 2.92E-05 |
| Kojibiose | HMDB0011742 | Lipids and lipid-like molecules | Fatty acyls | 4.882 | 6.73E-10 | 1.607 | 2.22E-06 | 3.22 (1.92, 5.42) | 9.85E-06 |
| Linoleic acid | HMDB0000673 | Lipids and lipid-like molecules | Fatty acyls | 0.504 | 9.25E-05 | 0.501 | 2.84E-09 | 0.46 (0.32, 0.65) | 1.17E-05 |
| Lithocholyltaurine | HMDB0000722 | Lipids and lipid-like molecules | Steroids and steroid derivatives | 0.632 | 5.07E-07 | 0.752 | 2.49E-05 | 0.38 (0.25, 0.58) | 6.17E-06 |
| Methyl jasmonate | HMDB0036583 | Lipids and lipid-like molecules | Fatty acyls | 0.572 | 4.01E-05 | 0.711 | 2.30E-04 | 0.51 (0.36, 0.71) | 8.97E-05 |
| MG(0:0/15:0/0:0) | HMDB0011532 | Lipids and lipid-like molecules | Glycerolipids | 0.523 | 6.14E-05 | 0.590 | 2.99E-05 | 0.51 (0.37, 0.71) | 5.35E-05 |
| Momordicilin | HMDB0030896 | Lipids and lipid-like molecules | Prenol lipids | 0.447 | 3.45E-06 | 0.645 | 1.66E-04 | 0.58 (0.43, 0.78) | 4.07E-04 |
| Momordol | Not available | Lipids and lipid-like molecules | Fatty acyls | 0.495 | 2.48E-10 | 0.609 | 7.20E-08 | 0.38 (0.25, 0.57) | 2.92E-06 |
| PE(16:0/18:1) | Not available | Lipids and lipid-like molecules | Glycerophospholipids | 3.221 | 2.35E-06 | 1.897 | 4.59E-02 | 1.71 (1.22, 2.40) | 1.80E-03 |
| PE(20:3e/3:0) | Not available | Lipids and lipid-like molecules | Glycerophospholipids | 0.435 | 2.36E-11 | 0.552 | 1.34E-10 | 0.33 (0.21, 0.51) | 4.40E-07 |
| PI(2:0/14:1) | Not available | Lipids and lipid-like molecules | Glycerophospholipids | 0.692 | 3.69E-03 | 0.655 | 4.43E-06 | 0.48 (0.34, 0.68) | 5.05E-05 |
| Reticulataxanthin | HMDB0036882 | Lipids and lipid-like molecules | Prenol lipids | 0.766 | 4.49E-02 | 0.620 | 1.21E-07 | 0.45 (0.31, 0.66) | 3.80E-05 |
| SQDG(12:0/13:0) | Not available | Lipids and lipid-like molecules | Glycerolipids | 0.684 | 2.87E-04 | 0.683 | 3.03E-03 | 0.55 (0.39, 0.79) | 9.20E-04 |
| 2-trans-6-cis-Dodecadienal | HMDB0032531 | Organic oxygen compounds | Organooxygen compounds | 0.466 | 7.54E-07 | 0.490 | 3.24E-09 | 0.45 (0.32, 0.64) | 7.25E-06 |
| Cer/AP(t14:2/13:1) | Not available | Organic oxygen compounds | Organooxygen compounds | 0.474 | 2.38E-11 | 0.609 | 7.26E-09 | 0.32 (0.21, 0.50) | 4.40E-07 |
| Cer/AP(t14:2/19:1) | Not available | Organic oxygen compounds | Organooxygen compounds | 0.621 | 1.76E-05 | 0.689 | 1.10E-04 | 0.56 (0.40, 0.79) | 8.68E-04 |
| Cer/AP(t15:1/16:2) | Not available | Organic oxygen compounds | Organooxygen compounds | 0.622 | 5.53E-06 | 0.592 | 1.06E-06 | 0.45 (0.32, 0.65) | 2.31E-05 |
| Cer/AP(t15:2/16:2) | Not available | Organic oxygen compounds | Organooxygen compounds | 0.658 | 1.29E-07 | 0.774 | 2.08E-05 | 0.31 (0.18, 0.52) | 9.41E-06 |
| Cer/AP(t15:2/18:2) | Not available | Organic oxygen compounds | Organooxygen compounds | 0.697 | 2.18E-04 | 0.551 | 3.47E-05 | 0.58 (0.42, 0.80) | 8.68E-04 |
| Cer/AP(t15:2/20:2) | Not available | Organic oxygen compounds | Organooxygen compounds | 0.690 | 5.65E-06 | 0.775 | 2.31E-03 | 0.61 (0.43, 0.85) | 3.71E-03 |
| Cer/AP(t17:2/12:1) | Not available | Organic oxygen compounds | Organooxygen compounds | 0.663 | 3.17E-07 | 0.799 | 9.15E-05 | 0.36 (0.22, 0.57) | 1.89E-05 |
| Cer/AS(d14:2/13:1) | Not available | Organic oxygen compounds | Organooxygen compounds | 0.533 | 3.21E-10 | 0.591 | 6.30E-09 | 0.25 (0.14, 0.42) | 4.80E-07 |
| Cer/AS(d14:3/13:1) | Not available | Organic oxygen compounds | Organooxygen compounds | 0.511 | 7.43E-11 | 0.691 | 3.34E-06 | 0.43 (0.30, 0.63) | 1.07E-05 |
| Cer/AS(d15:3/16:2) | Not available | Organic oxygen compounds | Organooxygen compounds | 0.719 | 7.22E-04 | 0.797 | 3.76E-02 | 0.59 (0.43, 0.82) | 1.62E-03 |
| D-Maltose | HMDB0000163 | Organic oxygen compounds | Organooxygen compounds | 4.617 | 2.36E-11 | 1.556 | 3.07E-06 | 2.97 (1.84, 4.82) | 9.62E-06 |
| Methyl lucidenate F | HMDB0036437 | Organic oxygen compounds | Prenol lipids | 0.546 | 4.28E-08 | 0.718 | 3.13E-03 | 0.52 (0.36, 0.74) | 3.18E-04 |
| Raffinose | HMDB0003213 | Organic oxygen compounds | Organooxygen compounds | 4.793 | 3.76E-08 | 1.746 | 4.43E-06 | 2.34 (1.53, 3.59) | 8.95E-05 |
| Trehalose | HMDB0000975 | Organic oxygen compounds | Organooxygen compounds | 3.580 | 2.38E-11 | 2.557 | 1.24E-06 | 2.44 (1.59, 3.75) | 4.55E-05 |
| (10)-Gingerol | HMDB0033616 | Benzenoids | Phenols | 0.640 | 5.50E-07 | 0.675 | 8.46E-04 | 0.43 (0.28, 0.65) | 6.48E-05 |
| [12]-Gingerol | HMDB0036356 | Benzenoids | Phenols | 0.597 | 3.64E-11 | 0.717 | 1.09E-05 | 0.28 (0.17, 0.47) | 6.90E-07 |
| [2,2-Bis(2-methylpropoxy)ethyl]benzene | HMDB0037712 | Benzenoids | Benzene and substituted derivatives | 0.338 | 2.86E-10 | 0.522 | 1.27E-08 | 0.37 (0.25, 0.56) | 1.42E-06 |
| 2-Methyl-1-phenyl-2-propanyl butyrate | HMDB0040226 | Benzenoids | Benzene and substituted derivatives | 0.506 | 2.36E-11 | 0.596 | 7.73E-06 | 0.32 (0.20 0.49) | 5.10E-07 |
| 3,4-Dihydroxybenzoic acid | HMDB0001856 | Benzenoids | Benzene and substituted derivatives | 0.423 | 2.62E-10 | 0.649 | 1.94E-02 | 0.49 (0.35, 0.70) | 6.35E-05 |
| Ginkgoic acid | HMDB0033897 | Benzenoids | Benzene and substituted derivatives | 0.741 | 2.07E-03 | 0.741 | 2.84E-04 | 0.48 (0.32, 0.72) | 3.20E-04 |
| Alanyl-Arginine | HMDB0028681 | Organic acids and derivatives | Carboxylic acids and derivatives | 0.731 | 1.04E-03 | 0.626 | 6.93E-11 | 0.22 (0.12, 0.39) | 2.90E-07 |
| Isomultiflorenyl acetate | HMDB0038062 | Organic acids and derivatives | Carboxylic acids and derivatives | 0.419 | 2.36E-11 | 0.612 | 7.66E-09 | 0.35 (0.23, 0.52) | 4.20E-07 |
| LDGTS(16:4) | LipidBlast2022_444989 | Organic acids and derivatives | Carboxylic acids and derivatives | 0.576 | 2.72E-06 | 0.730 | 3.19E-03 | 0.57 (0.41, 0.79) | 8.64E-04 |
| N-Salicyloylaspartic acid | HMDB0039506 | Organic acids and derivatives | Carboxylic acids and derivatives | 0.515 | 5.86E-05 | 0.408 | 6.24E-11 | 0.14 (0.06, 0.32) | 3.51E-06 |
| 1-Pyrroline-2-carboxylic acid | HMDB0006875 | Organoheterocyclic compounds | Pyrrolines | 0.792 | 1.15E-02 | 0.702 | 4.29E-07 | 0.55 (0.40, 0.76) | 3.35E-04 |
| 2-Hydroxyxanthone | HMDB0032997 | Organoheterocyclic compounds | Benzopyrans | 0.763 | 1.20E-03 | 0.768 | 1.19E-03 | 0.67 (0.48, 0.93) | 1.55E-02 |
| Furanone A | HMDB0094691 | Organoheterocyclic compounds | Dihydrofurans | 1.246 | 3.00E-03 | 1.271 | 6.30E-04 | 1.67 (1.18, 2.36) | 3.66E-03 |
| Phaeophorbide b | HMDB0031149 | Organoheterocyclic compounds | Tetrapyrroles and derivatives | 0.761 | 1.03E-02 | 0.521 | 6.24E-11 | 0.56 (0.40, 0.80) | 1.24E-03 |
| xi-5-Dodecanolide | HMDB0037742 | Organoheterocyclic compounds | Lactones | 0.509 | 2.28E-06 | 0.576 | 2.36E-06 | 0.50 (0.35, 0.69) | 4.11E-05 |
| Adenosine 3',5'-diphosphate | HMDB0000061 | Nucleosides, nucleotides, and analogues | Purine nucleotides | 0.593 | 8.84E-05 | 0.604 | 1.42E-07 | 0.49 (0.34, 0.71) | 2.06E-04 |
| ADP | HMDB0001341 | Nucleosides, nucleotides, and analogues | Purine nucleotides | 0.664 | 2.24E-04 | 0.583 | 2.44E-08 | 0.43 (0.27, 0.68) | 3.51E-04 |
| Kuwanon V | HMDB0030115 | Phenylpropanoids and polyketides | Diarylheptanoids | 0.715 | 1.08E-02 | 0.353 | 6.24E-11 | 0.43 (0.29, 0.66) | 7.19E-05 |
| Mulberrofuran E | HMDB0041423 | Phenylpropanoids and polyketides | 2-arylbenzofuran flavonoids | 0.597 | 8.49E-05 | 0.400 | 6.24E-11 | 0.34 (0.20, 0.55) | 1.36E-05 |

^†^ ANOVA with Tukey’s honestly significant difference tests was used to calculate P values, and false discovery rate estimation was applied for multiple testing correction.

^‡^ Conditional regression models were used to calculate odds ratios per 1-SD increment of natural-log transformed metabolite concentrations in the combined dataset, with adjustment for age (years), area (Nanjing, Guangzhou), body mass index (kg/m^2^), smoking status (current, non-current), and alcohol drinking (current, non-current).

Abbreviations: FDR, false discovery rate; OR, Odds Ratio; CRC, colorectal cancer; NC, normal control.

| **Table S2.** The differential metabolites within the top three pathways | |
| --- | --- |
| **Pathway** | **Differential** **metabolite** |
| **CRC vs. NC** |  |
| Alpha Linolenic Acid and Linoleic Acid Metabolism | Linoleic acid |
|  | Stearidonic acid |
| Threonine and 2-Oxobutanoate Degradation | 2-Ketobutyric acid |
|  | Adenosine diphosphate |
| Sulfate/Sulfite Metabolism | Adenosine 3',5'-diphosphate |
|  | Adenosine diphosphate |
| **CRA vs. NC** |  |
| Glycerolipid Metabolism | Palmitic acid |
| Fatty Acid Elongation in Mitochondria | Palmitic acid |
| Fatty Acid Biosynthesis | Palmitic acid |
| **CRC vs. CRA** |  |
| Trehalose Degradation | Trehalose |
|  | Adenosine diphosphate |
| Sulfate/Sulfite Metabolism | Adenosine 3',5'-diphosphate |
|  | Adenosine diphosphate |
| Bile Acid Biosynthesis | Cholic acid |
|  | Lithocholyltaurine |
|  | Adenosine diphosphate |
| Abbreviations: CRA, colorectal adenoma; CRC, colorectal cancer; NC, normal control. | |

**Table S3.** The performance of individual metabolites to distinguish colorectal cancer, colorectal adenoma, and normal control

| **Metabolite** | **Superclass** | **Class** | **AUC (95% CI)^†^** | |
| --- | --- | --- | --- | --- |
|  |  |  | **SVM** | **Logistic regression** |
| **CRC vs. NC** |  |  |  |  |
| (9S,10E,12Z,15Z)-9-Hydroxy-10,12,15-octadecatrienoic acid | Lipids and lipid-like molecules | Lineolic acids and derivatives | 0.644 (0.567-0.721) | 0.635 (0.557-0.713) |
| Carnosic acid | Lipids and lipid-like molecules | Prenol lipids | 0.651 (0.572-0.729) | 0.650 (0.572-0.728) |
| DGTS(2:0/20:1) | Lipids and lipid-like molecules | Glycerolipids | 0.562 (0.486-0.637) | 0.508 (0.432-0.584) |
| LPC(19:0) | Lipids and lipid-like molecules | Glycerophospholipids | 0.727 (0.654-0.800) | 0.728 (0.655-0.800) |
| LysoPI(18:0/0:0) | Lipids and lipid-like molecules | Glycerophospholipids | 0.593 (0.514-0.671) | 0.480 (0.399-0.560) |
| Methyl jasmonate | Lipids and lipid-like molecules | Fatty acyls | 0.567 (0.486-0.648) | 0.515 (0.433-0.597) |
| 3,4-Dihydroxybenzoic acid | Benzenoids | Benzene and substituted derivatives | 0.664 (0.588-0.741) | 0.634 (0.555-0.713) |
| 2-Hydroxyxanthone | Organoheterocyclic compounds | Benzopyrans | 0.587 (0.507-0.666) | 0.578 (0.499-0.658) |
| Cer/AS(d14:2/13:1) | Organic oxygen compounds | Organooxygen compounds | 0.724 (0.650-0.798) | 0.755 (0.686-0.823) |
| 4-Methyl-2-oxovaleric acid | Organic acids and derivatives | Keto acids and derivatives | 0.601 (0.523-0.680) | 0.488 (0.406-0.570) |
| **CRA vs. NC** |  |  |  |  |
| (9S,10E,12Z,15Z)-9-Hydroxy-10,12,15-octadecatrienoic acid | Lipids and lipid-like molecules | Lineolic acids and derivatives | 0.596 (0.522-0.670) | 0.584 (0.509-0.659) |
| ACar(18:3) | Lipids and lipid-like molecules | Fatty acyls | 0.576 (0.501-0.651) | 0.536 (0.460-0.612) |
| Palmitoylethanolamide | Organic acids and derivatives | Carboximidic acids and derivatives | 0.565 (0.489-0.640) | 0.533 (0.457-0.608) |
| Phytosphingosine | Organic nitrogen compounds | Organonitrogen compounds | 0.478 (0.402-0.554) | 0.492 (0.416-0.568) |
| PI(16:0/18:3) | Lipids and lipid-like molecules | Glycerophospholipids | 0.637 (0.564-0.710) | 0.645 (0.573-0.717) |
| Setariol | Lipids and lipid-like molecules | Steroids and steroid derivatives | 0.607 (0.533-0.682) | 0.572 (0.497-0.648) |
| SHexCer(d29:1) | Lipids and lipid-like molecules | Fatty acyls | 0.843 (0.789-0.896) | 0.858 (0.809-0.907) |
| **CRC vs. CRA** |  |  |  |  |
| (3beta,5alpha,6beta,7alpha,22E,24R)-Ergosta-8,22-diene-3,5,6,7-tetrol | Lipids and lipid-like molecules | Steroids and steroid derivatives | 0.747 (0.696-0.798) | 0.770 (0.723-0.818) |
| ACar(10:0) | Lipids and lipid-like molecules | Fatty acyls | 0.709 (0.656-0.761) | 0.727 (0.677-0.777) |
| Cer/AP(t14:2/13:1) | Organic oxygen compounds | Organooxygen compounds | 0.733 (0.679-0.787) | 0.762 (0.713-0.810) |
| Mulberrofuran E | Phenylpropanoids and polyketides | 2-arylbenzofuran flavonoids | 0.763 (0.715-0.811) | 0.769 (0.722-0.816) |
| (R)-3-Hydroxy-tetradecanoic acid | Lipids and lipid-like molecules | Fatty acyls | 0.701 (0.647-0.755) | 0.726 (0.676-0.777) |
| Trehalose | Organic oxygen compounds | Organooxygen compounds | 0.719 (0.668-0.771) | 0.754 (0.706-0.802) |

^†^ AUC was calculated by SVM and logistic regression.

Abbreviations: AUC, area under the receiver operating characteristic curve; SVM, support vector machine; CRC, colorectal cancer; NC, normal control; LPC: lysophosphatidylcholine; CRA, colorectal adenoma.

**Table S4.** Demographic characteristics of participants in the Nanjing, Guangzhou, and Kunming studies

| **Variable** | **Nanjing study** | | | | **Guangzhou study** | | | | **Kunming study** | | | |
| --- | --- | --- | --- | --- | --- | --- | --- | --- | --- | --- | --- | --- |
|  | CRC | CRA | NC | *P* value^†^ | CRC | CRA | NC | *P* value^†^ | CRC | CRA | NC | *P* value^†^ |
|  | (n=112) | (n=57) | (n=112) |  | (n=107) | (n=107) | (n=107) |  | (n=91) | (n=115) | (n=109) |  |
| Age, mean (SD), years | 59.9 (8.3) | 62.8 (9.3) | 58.7 (8.6) | 0.02 | 55.8 (7.6) | 56.7 (7.4) | 56.4 (7.9) | 0.67 | 62.1 (11.7) | 54.9 (11.0) | 43.3 (11.6) | <0.001 |
| Gender, No. (%) |  |  |  |  |  |  |  |  |  |  |  |  |
| Male | 57 (50.9) | 35 (61.4) | 57 (50.9) | 0.37 | 64 (59.8) | 64 (59.8) | 64 (59.8) | 1.00 | 54 (59.3) | 73 (63.5) | 45 (41.3) | 0.002 |
| Female | 55 (49.1) | 22 (38.6) | 55 (49.1) |  | 43 (40.2) | 43 (40.2) | 43 (40.2) |  | 37 (40.7) | 42 (36.5) | 64 (58.7) |  |
| Body mass index, mean (SD), kg/m^2^ | 23.8 (3.2) | 24.2 (3.6) | 23.3 (2.9) | 0.16 | 23.0 (2.9) | 23.3 (3.0) | 23.2 (2.7) | 0.81 | 22.1 (3.0) | 23.3 (3.3) | 21.9 (3.1) | 0.002 |
| Smoking status, No. (%)^‡^ |  |  |  |  |  |  |  |  |  |  |  |  |
| Non-current | 86 (76.8) | 39 (68.4) | 89 (79.4) | 0.28 | 80 (74.8) | 52 (48.6) | 87 (81.3) | 0.02 | 66 (73.4) | 80 (69.6) | 91 (83.5) | 0.05 |
| Current | 26 (23.2) | 18 (31.6) | 23 (20.5) |  | 27 (25.2) | 30 (28.0) | 20 (18.7) |  | 24 (26.7) | 35 (30.4) | 18 (16.5) |  |
| Alcohol drinking, No. (%)^‡^ |  |  |  |  |  |  |  |  |  |  |  |  |
| Non-current | 87 (77.7) | 40 (70.2) | 94 (83.9) | 0.11 | 88 (82.2) | 63 (58.9) | 77 (72.0) | 0.01 | 73 (81.1) | 93 (80.9) | 98 (89.9) | 0.12 |
| Current | 25 (22.3) | 17 (29.8) | 18 (16.1) |  | 19 (17.8) | 19 (17.8) | 30 (28.0) |  | 17 (18.9) | 22 (19.1) | 11 (10.1) |  |

^†^ One-way ANOVA for continuous variables and chi-square tests for categorical variables.

^‡^ The totals did not sum to 100% in the Guangzhou study due to small proportions of participants did not answer the questions.

Abbreviations: CRC, colorectal cancer; CRA, colorectal adenoma; NC, normal control.
